# *In-Silico* Analysis of Inflammatory Bowel Disease (IBD) GWAS Loci to Novel Connections

**DOI:** 10.1371/journal.pone.0119420

**Published:** 2015-03-18

**Authors:** Md. Mesbah-Uddin, Ramu Elango, Babajan Banaganapalli, Noor Ahmad Shaik, Fahad A. Al-Abbasi

**Affiliations:** 1 Department of Biochemistry, Faculty of Science, King Abdulaziz University, Jeddah, Saudi Arabia; 2 Princess Al-Jawhara Al-Brahim Center of Excellence in Research of Hereditary Disorders, King Abdulaziz University, Jeddah, Saudi Arabia; 3 Department of Genetic Medicine, Faculty of Medicine, King Abdulaziz University, Jeddah, Saudi Arabia; INSERM, FRANCE

## Abstract

Genome-wide association studies (GWASs) for many complex diseases, including inflammatory bowel disease (IBD), produced hundreds of disease-associated loci—the majority of which are noncoding. The number of GWAS loci is increasing very rapidly, but the process of translating single nucleotide polymorphisms (SNPs) from these loci to genomic medicine is lagging. In this study, we investigated 4,734 variants from 152 IBD associated GWAS loci (IBD associated 152 lead noncoding SNPs identified from pooled GWAS results + 4,582 variants in strong linkage-disequilibrium (LD) (*r^2^* ≥0.8) for EUR population of 1K Genomes Project) using four publicly available bioinformatics tools, e.g. dbPSHP, CADD, GWAVA, and RegulomeDB, to annotate and prioritize putative regulatory variants. Of the 152 lead noncoding SNPs, around 11% are under strong negative selection (GERP++ RS ≥2); and ~30% are under balancing selection (Tajima’s D score >2) in CEU population (1K Genomes Project)—though these regions are positively selected (GERP++ RS <0) in mammalian evolution. The analysis of 4,734 variants using three integrative annotation tools produced 929 putative functional SNPs, of which 18 SNPs (from 15 GWAS loci) are in concordance with all three classifiers. These prioritized noncoding SNPs may contribute to IBD pathogenesis by dysregulating the expression of nearby genes. This study showed the usefulness of integrative annotation for prioritizing fewer functional variants from a large number of GWAS markers.

## Introduction

Inflammatory bowel disease (IBD), mainly classified as Crohn’s disease (CD) and Ulcerative colitis (UC), is one of the major immune-mediated inflammatory conditions of the alimentary tract, which affected around 2.5 million European [1], 1.8 million adult Americans [2], with increasing prevalence in Asia and other developing countries where people are embracing Westernized lifestyle and food habit [3–5]. The incidence is also increasing in children around the world due to prenatal and postnatal exposures to various risk factors [6]. The pathogenesis of IBD is largely attributed to environmental, immunologic and genetic factors [7, 8], where host genome and mucosal immune cells interact with gut microbiota along with other environmental stimuli [9, 10]. However, it is difficult to follow a unified regime for treating IBD owing to the inherent disease heterogeneity [11, 12], which demands patient stratification based on risk factors and development of tailored medicine [12]. Though the treatment of inflammatory diseases witnessed a rapid advancement in the last decade and immunotherapy showed success in ameliorating inflammatory conditions, efficacy is still an issue for treating IBD patients [12]. Besides, the cost related to anti-TNFα therapy [13], polypharmacy (mainly analgesic and psychiatric drugs) among IBD patients [14], and productivity losses due to work disability [15], are worsening the situation as well as increasing economic burden.

The research of human genetics and personalized medicine has advanced remarkably in the advent of high throughput genomic techniques. Over the last decade, hundreds of trait associated variants were identified through genome-wide association studies (GWASs) in diverse populations, which reinforced our understanding of complex traits including height, weight, diabetes, cancer, and immune-mediated diseases, such as rheumatoid arthritis (RA), and multiple sclerosis (MS) [16–18]. More notably, GWAS proved to be very successful in identifying IBD susceptibility loci and related pathways. The recent meta-analysis of GWAS findings and subsequent extensive validation of the signals in European population have brought the IBD associated risk loci to 163—highest for any single disease [1]. However, very few of these GWASs variants contributed in translational medicine for early diagnosis and treatment [19]. This could be due to the challenge of assigning relevant biological information to associated noncoding regions (around 90% of GWAS variants) [20] and pinpointing causal variants from the GWAS loci [21, 22]. Though individual risk locus accounts for modest effect in complex diseases [17], the need for exploring molecular mechanisms through the identification of functional variants from the GWAS signals is immense [23]. However, putative functional variants could be distinguished from the GWAS loci through integrative annotation and prioritization of the variants [24, 25]. In this study, we analyzed IBD associated lead noncoding single-nucleotide polymorphisms (SNPs) from a meta-analysis of 15 GWASs on European population [1], along with variants in strong linkage-disequilibrium (LD) (*r* ^*2*^ ≥0.8) with the lead SNPs, using publicly available functional annotation tools, to prioritize regulatory variants from the GWAS loci and deduce probable biological link to IBD pathogenesis.

## Methods

### Dataset

IBD associated 163 lead single-nucleotide polymorphisms (SNPs) from Jostins et al. [1] report were considered as the core dataset in this study and relevant information were extracted from *NHGRI GWAS Catalog* [16]. Among the 163 lead SNPs, 152 SNPs (~93%) are in noncoding regions and 11 SNPs (~7%) are in coding regions of the genome. Of the eleven coding SNPs, 7 are nonsynonymous SNPs (nsSNPs), 3 synonymous SNPs and one frameshift mutation on *NOD2* gene. However, in this study we only focused on the 152 lead noncoding SNPs and variants in strong linkage disequilibrium (LD) (*r* ^*2*^ ≥0.8) with these SNPs to identify regulatory variants from the studied 152 GWAS loci. Reference SNP cluster IDs (rsIDs) and *r* ^*2*^ values for LD variants were extracted from HaploReg v2 [26] web server (http://www.broadinstitute.org/mammals/haploreg/haploreg.php, accessed December 18, 2013) for EUR (European) population of 1K Genomes Project (1KGP). Chromosome positions of all SNPs are based on human genome assembly GRCh37 (hg19). There are three main categories of mutations in our dataset, e.g. single-nucleotide polymorphisms (SNPs), insertions and deletions (indels). To reduce ambiguity, the term SNP and variant was used interchangeably throughout the article.

### Selective Constraints

We analyzed 152 lead noncoding SNPs to identify regions under selective constraints, using evolutionary conservation statistic, e.g. position-specific neutral rate (NR) and rejected substitution (RS) scores (*RS = Neutral rate (NR)—Observed substitution*) from Genomic Evolutionary Rate Profiling (GERP++) [27, 28], and population specific statistics, e.g. Tajima’s D (TD) score [29], derived allele frequency (DAF) and difference of derived allele frequency (ΔDAF) between queried population and all other populations. GERP++ RS and NR, TD, DAF and ΔDAF scores were retrieved from the webserver of dbPSHP [30] (http://jjwanglab.org/dbpshp, last accessed February 20, 2014) using default settings. The CEU population (Utah Residents with Northern and Western European ancestry) of 1KGP is the queried population for TD, DAF and ΔDAF.

### Annotation Tools

IBD associated lead noncoding SNPs and variants in strong LD with lead SNPs were analyzed using three publicly available annotation tools, e.g. Combined Annotation-Dependent Depletion (CADD) [24], Genome Wide Annotation of Variants (GWAVA) [25] and RegulomeDB [31].

#### CADD

Combined Annotation-Dependent Depletion (CADD) is a support vector machine trained online prediction tool for prioritizing functional, deleterious and pathogenic variants from diverse categories of variants [24]. For each query variant, CADD provides a combined annotation score (C-score) from its comprehensive database of pre-computed C-scores for 8.6 billion possible human SNPs. According to CADD classification, top 10% probable functional variants will have a C-score of 10 or greater (≥C10), top 1% variants will have a C-score of 20 or greater (≥C20) and finally most deleterious top 0.1% human variants will have a C-score of 30 or greater (≥C30). For our study we extracted CADD scores for lead and LD variants from the webserver (http://cadd.gs.washington.edu, last accessed March 12, 2014) using prescribed variant call format (VCF).

#### GWAVA

Genome Wide Annotation of Variants (GWAVA) is a random forest trained online tool for predicting the functional impact of noncoding variants based on integration of different genomic and epigenomic annotations [25]. For each SNP, GWAVA provides three scores by three different versions of the classifier e.g. Region score, TSS score (based on nearest transcription start site) and Unmatched score; and the score ranges from zero to one—where higher scores indicate variants more likely to be functional. However, here we only considered TSS score as our GWAVA score, because it incorporates various regulatory annotations and is less dominated by any single annotation. We retrieved pre-computed GWAVA scores and annotations for the IBD associated noncoding SNPs and LD variants from the webserver (http://www.sanger.ac.uk/sanger/StatGen_Gwava, last accessed March 12, 2014) for further analysis.

#### RegulomeDB

RegulomeDB is an online database and prediction tool to annotate and prioritize potential regulatory variants from human genome [31]. The database includes high-quality datasets from Encyclopedia of DNA Elements (ENCODE) [32] and other sources, as well as predicted and manually curated annotations to identify putative regulatory variants. RegulomeDB classifies variants into six main categories (from Category 1 to Category 6), where Category 1 variants are “likely to affect binding and linked to expression of a gene target”, Category 2 variants are “likely to affect binding”, Category 3 variants are “less likely to affect binding”, and Category 4, 5 & 6 variants have “minimal binding evidence” [31]. RegulomeDB also assigns a score of 7 for variants with no annotation data available. However, we downloaded RegulomeDB annotations for lead noncoding SNPs and strong LD variants from the webserver (http://regulome.stanford.edu, last accessed May 19, 2014).

### Data Analysis, Visualization and Inference

For producing scatterplots, histograms, boxplots, bar graph and Venn diagram, *R* core package (version 3.0.3) [33] along with other *R* packages e.g. *ggplot2* [34] and *VennDiagram* [35] were used. The gene-interaction network is drawn providing all the genes nearby the prioritized SNPs to the GeneMANIA [36] webserver (http://www.genemania.org), and highly connected gene clusters were identified and visualized using Cytoscape 2.8.2 [37] and its plugin MCODE v1.32 [38]. *Ensembl Variant Effect Predictor (VEP)* [39] is used for obtaining the genes nearby the prioritized SNPs.

Two-sided Wilcoxon rank sum test (using *R* function *wilcox*.*test*) is conducted to show whether there is a significant difference in the distribution of annotation scores, from CADD and GWAVA tools, between IBD associated lead noncoding GWAS SNPs and variants in strong LD with the lead SNPs.

We calculated fold-enrichment for genes in a disease of interest as *((m/n)/(M/N))*, where *m* is the number of prioritized genes (using a tool or a combination of tools) belongs to the disease of interest, *n* is the total number of genes prioritized using a tool (or combination of tools), *M* is the total number of genes belong to the disease of interest, and *N* is the human genome background (30,000 genes). We applied Fisher’s exact test (using *R* function *fisher*.*test*) to estimate the statistical significance of each enrichment score. For this analysis, we considered genome-wide association studies (GWASs) of six immune-mediated diseases (IMDs) and retrieved the reported genes (HGNC [40] approved gene symbols, downloaded from www.genenames.org, on May 8, 2014) from these studies—ankylosing spondylitis (AS) [16, 41], celiac disease (CeD) [16], inflammatory bowel disease (IBD) [1], psoriasis (Ps) [16, 42, 43], rheumatoid arthritis (RA) [16], and type 1 diabetes (T1D) [16]. GWASs related data were obtained from the *NHGRI GWAS Catalog* (unless otherwise referred) (http://www.genome.gov/gwastudies) [16] on September 26, 2014; and *VEP* is used to obtain genes nearby the prioritized SNPs (only HGNC approved gene symbols were counted for the test). We also estimated the enrichment scores and *p-values* combining all the genes (after removing the duplicates) from four reference IMDs (IMD_4_: AS+CeD+Ps+T1D), as well as five reference diseases (IMD_5_: AS+CeD+Ps+RA+T1D).

And finally, the strength of correlation between the top 18 prioritized SNPs and lead SNPs from six IMDs e.g. AS [16, 41], CeD [16], IBD [1], Ps [16, 42, 43], RA [16] and T1D [16] were checked for European population of the 1K Genomes Project. We retrieved all the lead SNPs for the six IMDs from *NHGRI GWAS catalog* (and literatures), and searched in HaploReg v2 webserver [26] to identify the corresponding (strong) LD variants. We then manually screened the LD variant lists, for each reference disease, to identify the overlap with the top 18 prioritized SNPs.

## Results

### Selective constraints on IBD loci

**Fig 1:**
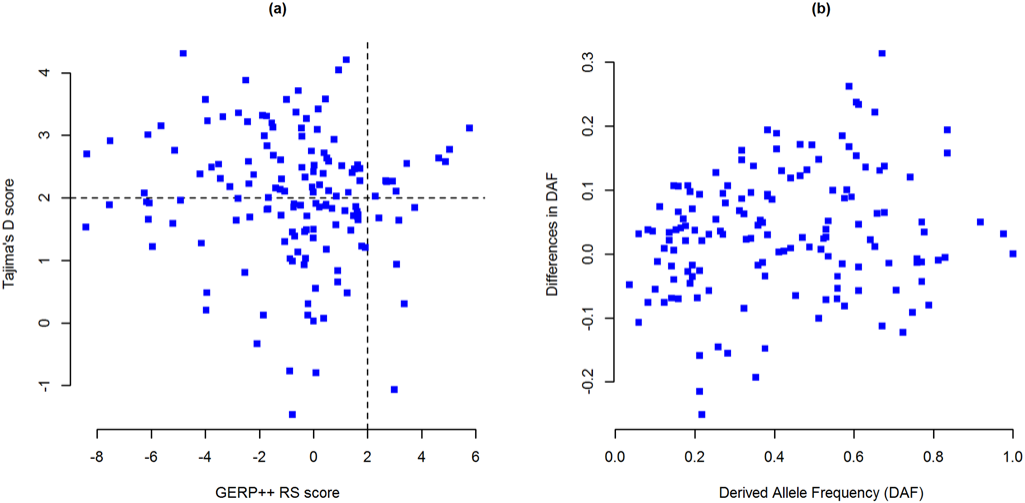
Selective constraints on IBD associated lead noncoding SNPs. (**a**) Evolutionary conservation versus human specific selection on lead SNPs. Evolutionary conservation is presented by GERP++ RS scores (*x axis*) and human specific selection is presented by Tajima’s D scores (*y axis*). Each point represents a single SNP; n = 148 SNPs. Positive GERP++ and TD scores indicate regions under negative selection and negative scores indicate positive selection. SNPs in the top-right segment are under strong evolutionary constraint as well as human specific selection. (**b**) Cross-population diversity of lead SNPs between CEU population and rest of the world. DAF is presented in the *x axis* and ΔDAF is presented in *y axis*. Each point represents a single SNP; n = 148 SNPs. **GERP++ RS**: Genomic Evolutionary Rate Profiling rejected substitution; **TD**: Tajima’s D; **DAF**: Derived allele frequency; **ΔDAF**: Difference in derived allele frequency; **CEU**: Utah residents with ancestry from Northern and Western Europe. TD, DAF and ΔDAF statistics are for 1K Genomes Project’s CEU population.

Of the 152 IBD associated lead noncoding variants, 65 SNPs have positive GERP++ RS scores (RS ≥0, i.e. substitution deficit) and 16 of these have very high RS scores (RS ≥2). In addition, 87 SNPs have negative RS scores (RS <0, i.e. substitution surplus)—among which 40 SNPs have RS scores less than 2 (Fig. 1a and S1 Table). The ratio of rejected substitution (RS) to neutral rate (NR) reveals 19 SNPs with no observed substitution (RS/NR = 1) in mammalian evolution (S1 Table) which could represent evolutionarily highly conserved positions.

We retrieved TD, DAF and ΔDAF scores from dbPSHP webserver for 148 SNPs (after removing 4 missing SNPs) and found that—in CEU population (1KGP), 143 IBD associated SNPs have positive TD scores and 5 SNPs have negative scores, where 77 of these SNPs have TD scores greater than 2 (TD >2) (Fig. 1a and S1 Table). Cross population diversity was presented in terms of derived allele frequencies (DAF) and differences in DAF (ΔDAF)—which showed that there are eight SNPs with a derived allele frequency greater than 0.8 (DAF ≥0.8) and 94 SNPs with higher derived allele frequency (ΔDAF >0) in CEU population than the rest of the world (Fig. 1b and S1 Table).

### Integrative Annotation of Lead and LD variants

We started our analysis with 152 lead noncoding SNPs and then extended to the surrounding variants in strong linkage-disequilibrium (LD) (*r* ^*2*^ ≥0.8) with the lead SNP. Using these criteria we found 4,582 strong LD variants for 143 lead SNPs in CEU population of 1KGP, whereas the remaining 9 lead SNPs are the only variants in the respective locus (S2 Table). The LD variants are composed of 4,308 single-nucleotide polymorphisms (SNPs), 203 deletions and 71 insertion mutation (total 4,582 LD variants). The insertions and deletions (indels) are mostly single nucleotide, though there are variants with 22-nucleotide deletion and 13-nucleotide insertion. However, we had CADD scores for all the variants, and RegulomeDB scores for the majority of the SNPs and few indels, but from GWAVA we only had scores for SNPs (no GWAVA score for indels). The functional annotation scores for 4,734 variants (152 lead + 4,582 LD variants) from the three classifiers e.g. CADD, GWAVA and RegulomeDB are discussed below.

#### Combined Annotation-Dependent Depletion (CADD)

**Fig 2:**
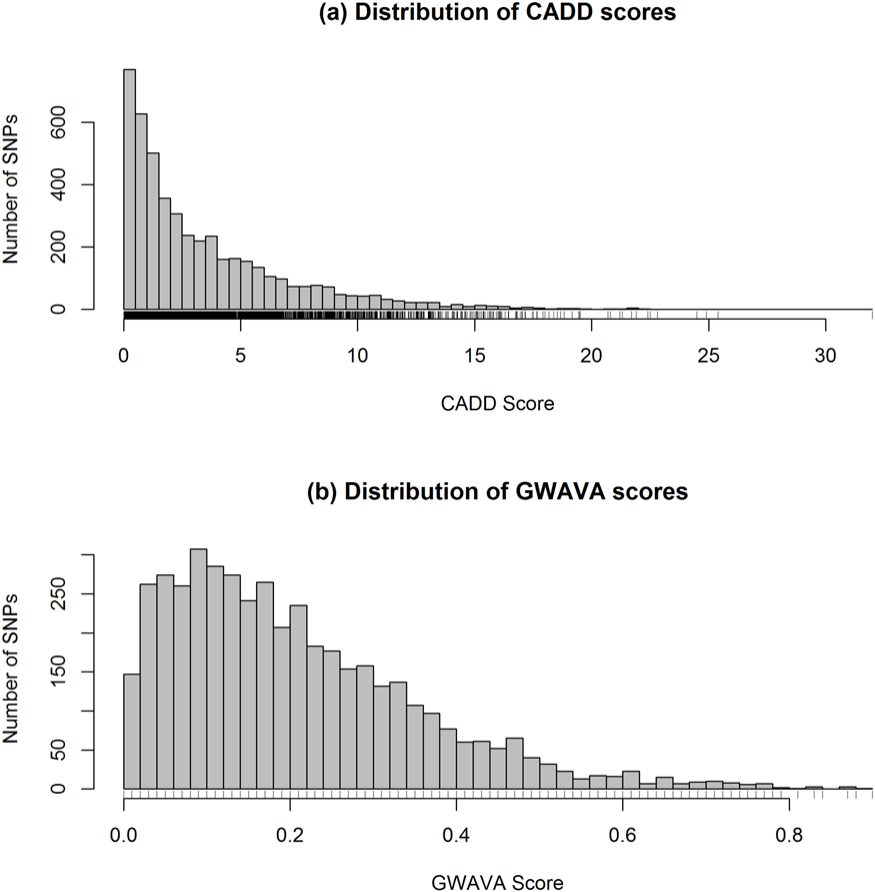
Distribution of (a) CADD and (b) GWAVA scores. Histograms are drawn taking CADD and GWAVA scores of all the variants (lead GWAS SNPs and LD variants) after removing the missing values. Here n = 4781 for CADD and n = 4460 variants for GWAVA.

**Fig 3:**
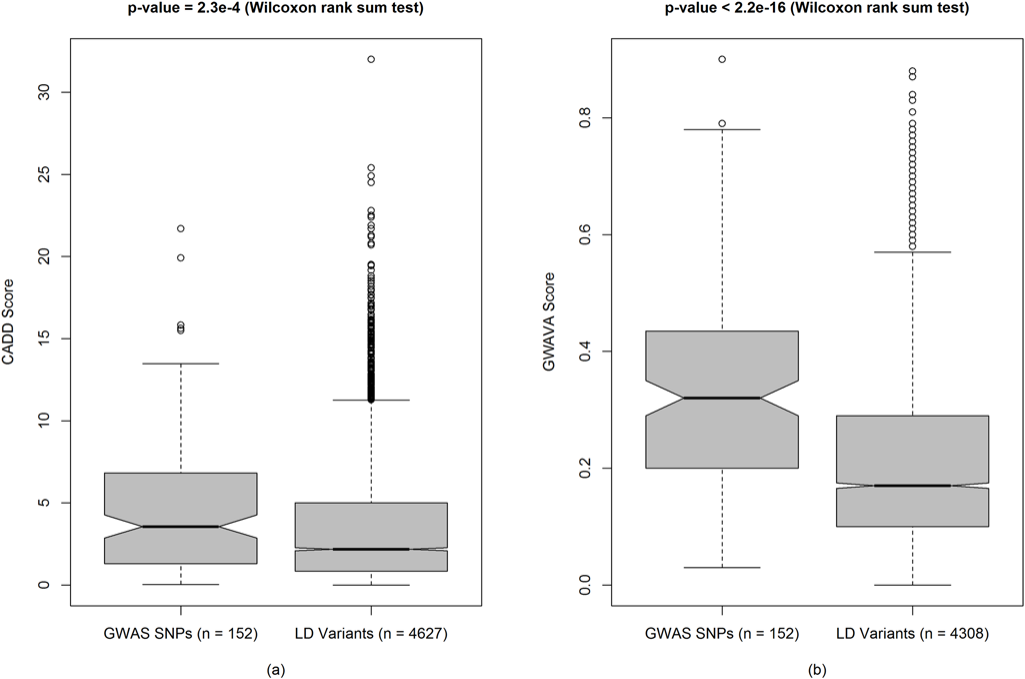
Comparing functional annotation scores of IBD associated lead noncoding GWAS SNPs versus LD variants. (**a**) CADD scores for GWAS (n = 152) versus LD variants (n = 4627). (**b**) GWAVA scores for GWAS (n = 152) versus LD variants (n = 4308). In the boxplots, center lines show the medians of the values and box limits indicate the 25th & 75th percentiles (as determined by R software). Whiskers extend to 5th and 95th percentiles and outliers are represented by open circle dots. The notches are defined as ± 1.58 × IQR (interquartile range) / square root of n and represent the 95% confidence interval for each median (default in R software). *P-values* are calculated using two-sided Wilcoxon rank sum test.

From CADD we have 4,781 C scores for 4,734 variants—few more C-scores than the number of rsIDs, because CADD provides C-score for each substitution and some of the rsID represent more than one substitution (S2 Table). The distribution of CADD scores is presented in the histogram (Fig. 2a). Here CADD score ranges from 0 to 32 with a mean of 3.5 and median of 2.2. From the total of 4,734 variants, 323 have CADD scores greater than 10 (>C10), of which 14 variants have C-score greater than 20 (>C20), and only one variant scores greater than 30 (>C30). Overall top five variants, which are also top LD variants, are rs601338, rs74869386, rs34622044, rs2298428 and rs10891692 with a CADD score of 32, 25.4, 24.9, 24.5 and 22.8, respectively. The top ranked variant rs601338 (C-score of 32) is a stop gained mutant of *FUT2* gene which is in strong LD (*r* ^*2*^ = 1) with lead SNP rs516246. However, the top five lead GWAS SNPs, e.g. rs4256159, rs17229285, rs4246905, rs7608910 and rs3851228, had a C-score of 21.7, 19.91, 15.83, 15.62 and 15.49, respectively. Interestingly, 21 SNPs from 152 lead noncoding SNPs (~14%) had a C-score of 10 or higher against 302 SNPs from 4627 LD variants (~7%). Which indicates differences in the distribution of C-scores between lead GWAS SNPs and LD variants—where lead SNPs had higher annotation scores than variants in strong LD with the lead variants (*P-value* = 2.3 × 10^-4^, two-sided Wilcoxon rank sum test) (Fig. 3a).

#### Genome Wide Annotation of Variants (GWAVA)

Of the 4,734 variants analyzed, GWAVA returned scores for 4,460 variants (i.e. 274 variants missing) in the range of 0.0 to 0.9—with a mean of 0.21 and median of 0.18. The distribution of GWAVA scores for 4,460 variants (152 lead GWAS SNPs and 4,308 LD variants) is presented in Fig. 2b and detailed scores are presented in S2 Table. Here, 509 SNPs have a GWAVA score of 0.4 or greater (≥0.4)—among which 45 SNPs have a score ≥0.7. Overall, the top five SNPs are rs4728142, rs1698374, rs7559479, rs7603250 and rs1024611, with a GWAVA score of 0.9, 0.88, 0.87, 0.87 and 0.84, respectively. On the other hand, top five lead SNPs are rs4728142, rs9264942, rs4246215, rs917997 and rs1126510, with a GWAVA score of 0.9, 0.79, 0.78, 0.78 and 0.75, respectively. Interestingly, the first four lead GWAS SNPs are also found to have HGMD-Public [44] regulatory variant annotations, against only two of the top five LD variants (Table 1). However, we also observed differences in the distribution of GWAVA scores (as seen in CADD scores) between lead GWAS SNPs and strong LD variants (Fig. 3b)—on average, lead GWAS SNPs have higher annotation scores from GWAVA than the LD variants (*P-value* < 2.2 × 10^-16^, two-sided Wilcoxon rank sum test).

**Table 1:** List of SNPs with high GWAVA scores and HGMD-PUBLIC regulatory annotation.

Chromosome: Position	SNP	LD (*r* ^*2*^)	Lead SNP	GWAVA	HGMD-PUBLIC Annotation
**7:128573967**	rs4728142	1	rs4728142	0.9	CR084006: -8169 relative to initiation codon of *IRF5*
**6:31274380**	rs9264942	1	rs9264942	0.79	CR075248: -34532 relative to initiation codon of *HLA-C*
**11:61564299**	rs4246215	1	rs4246215	0.78	CR095633: +323 relative to termination codon of *FEN1*
**2:103070568**	rs917997	1	rs917997	0.78	CR084631: +1927 relative to termination codon of *IL18RAP*
**20:44746982**	rs1883832	0.98	rs1569723	0.81	CR052074: -1 relative to initiation codon of *CD40*
**17:32579788**	rs1024611	0.96	rs3091316	0.84	CR032718: -2508 relative to TSS of *CCL2*
**17:32582007**	rs2857656	0.96	rs3091316	0.83	CR090001: -362 relative to initiation codon of *CCL2*
**20:31367079**	rs1569686	0.88	rs4911259	0.75	CR076693: +16889 relative to transcription initiation site of *DNMT3B*
**19:55385435**	rs12462181	0.82	rs11672983	0.74	CR011063: -114 relative to TSS of *FCAR*

All the lead SNPs, as well as LD variants with GWAVA score ≥0.7 were manually screened for Human Gene Mutation Database (HGMD) [44] regulatory annotation (HGMD release 2013.3) using Ensembl Genome Browser [45]. **TSS**: Transcription start site.

#### RegulomeDB

**Fig 4:**
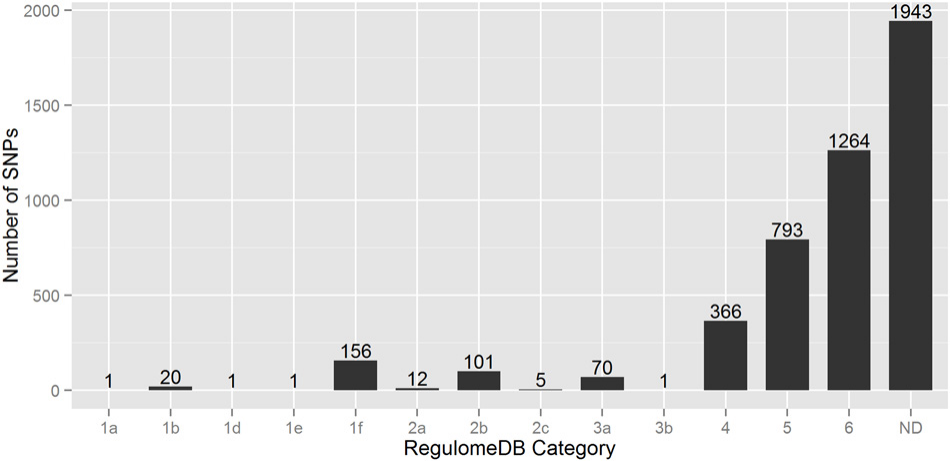
Bar diagram representing the number of SNPs in each RegulomeDB category. Here n = 4734 variants. **ND**: No Data.

In this scoring scheme, 4,734 SNPs are divided into six broad categories (Category 1 to Category 6)—where 2,791 SNPs had annotation scores in between 1 to 6, and the remaining 1,943 SNPs had no annotation data (Fig. 4). Of the 2,791 SNPs, 2,423 SNPs (~87%) had minimum functional evidence (Category 4, 5 & 6), 71 SNPs (~3%) are less likely to be functional (Category 3), and 297 SNPs (~10%) are more likely to have regulatory functions (Category 1 & 2). Importantly, around 18% (27 SNPs) of the lead GWAS SNPs had RegulomeDB score of less than 3, while only ~6% (270 SNPs) LD variants are in this list (S2 Table). However, it is surprising that from 297 putative regulatory SNPs only one SNP had RegulomeDB score of 1a. This top ranked SNP rs4788084 (in linkage-disequilibrium (*r* ^*2*^ = 0.82) with lead SNP rs26528) had annotation for eQTL, TF binding, matched TF motif, matched DNase footprint and DNase peak, and thus very likely to have regulatory functions. In contrast, the RegulomeDB score of 1f (eQTL + TF binding/DNase peak) turned out to be the common functional annotation for lead and LD variants—around 59% (16 of the 27 SNPs) lead SNPs and ~52% (140 of 270 SNPs) LD variants had an annotation score of 1f.

### Concordance Analysis

**Fig 5:**
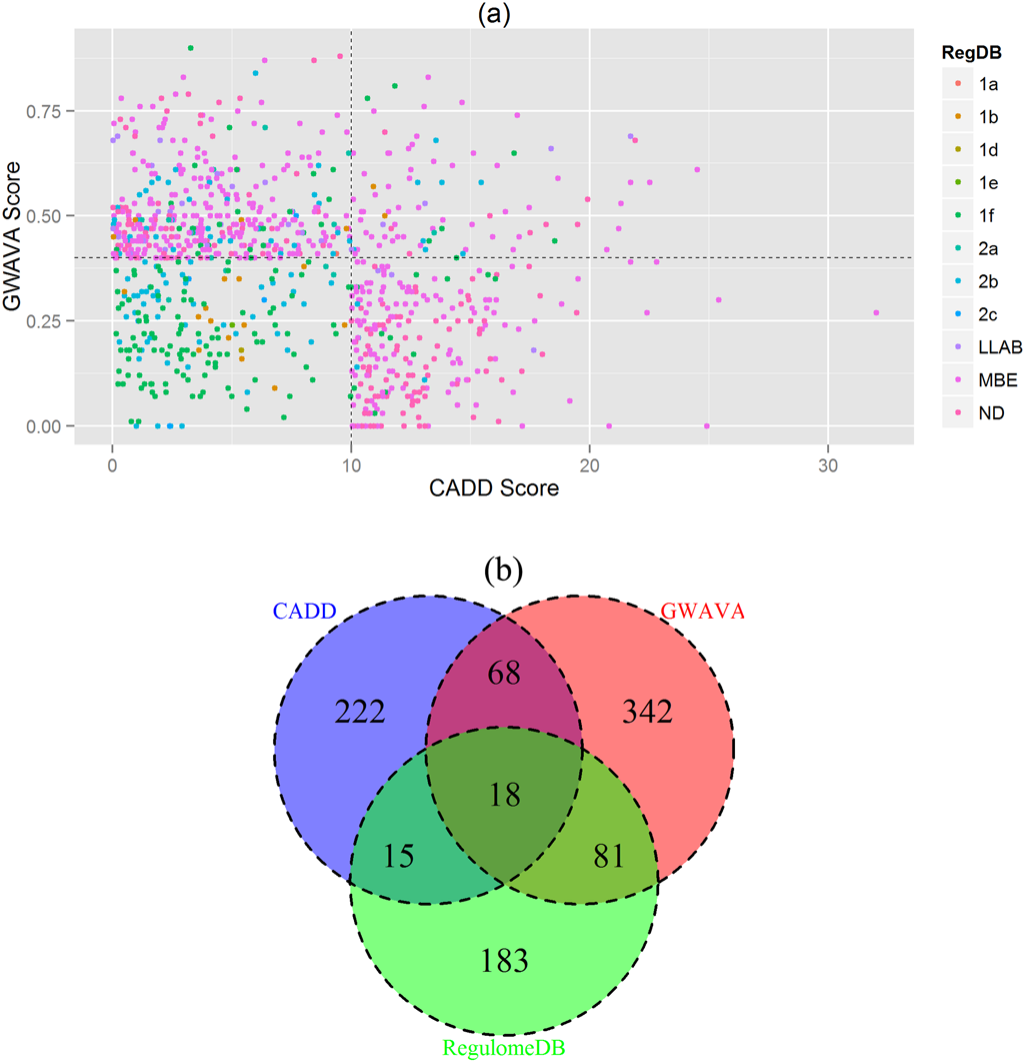
Concordance Analysis. (**a**) Scatterplot depicting top annotation scores from CADD, GWAVA and RegulomeDB. Each point represents a single SNP where CADD scores are in the *x axis*, GWAVA scores in the *y axis* and RegulomeDB annotation scores are shown with colors. The plot is divided into four segments using *y* intercept = 0.4 and *x* intercept = 10. Top right segment contains 18 SNPs with highest annotation scores from the three classifiers. (**b**) Venn diagram illustrating the number of SNPs in concordance with the three classifiers. Here CADD scores are in blue, GWAVA scores are in red and RegulomeDB scores are in green. The intersection of three circles represents SNPs in concordance with all three classifiers. Note: Cutoff scores used for concordance analysis are: CADD ≥10, GWAVA ≥0.4 and RegulomeDB ≤2. Based on this cutoff, 929 SNPs are plotted in the scatterplot and Venn diagram (after removing the overlap). **RegDB**: RegulomeDB annotation score; **LLAB**: Less likely to affect binding (includes category 3a and 3b); **MBE**: Minimal binding evidence (includes category 4, 5 and 6); **ND**: No Data.

**Fig 6:**
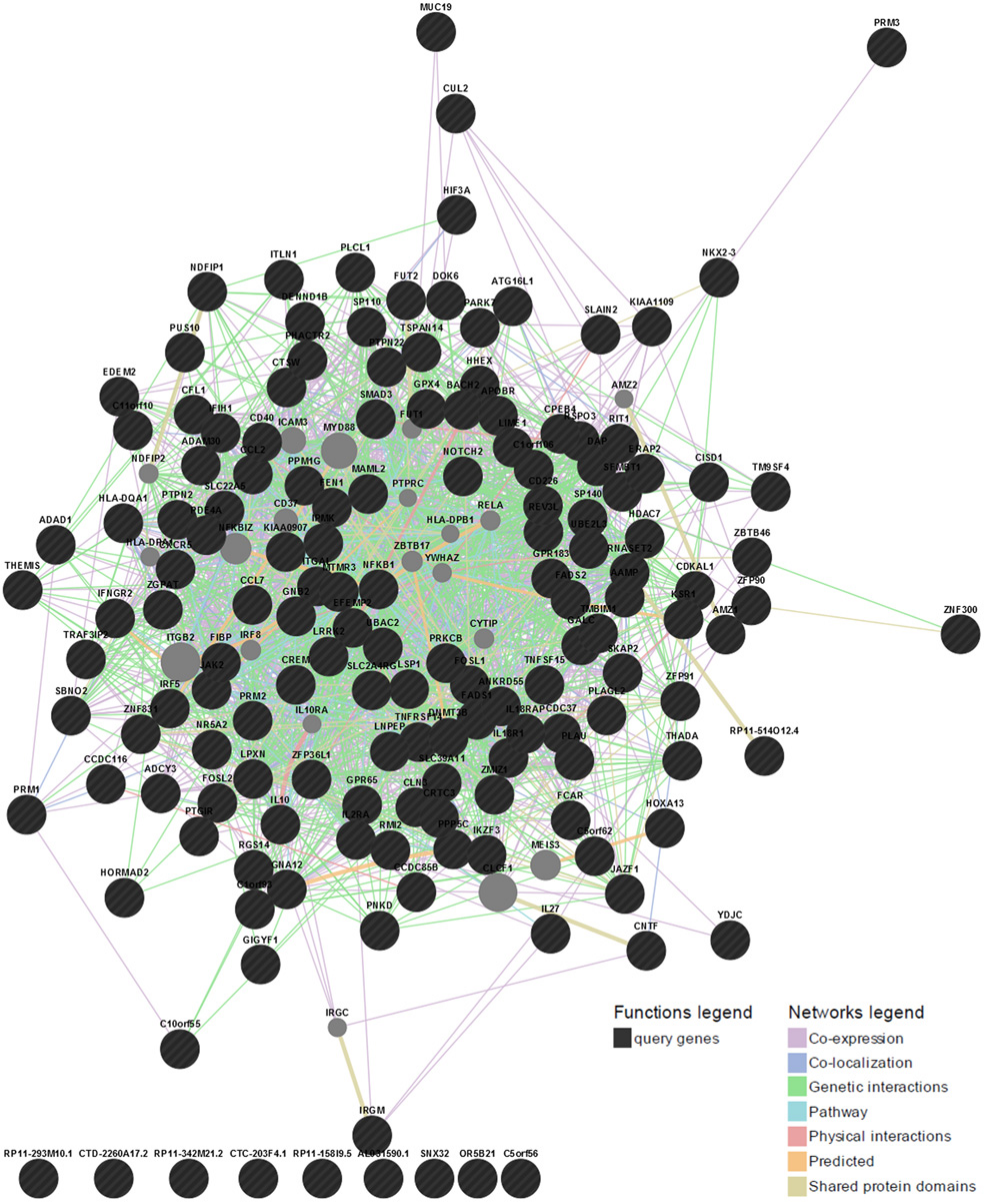
Gene interaction network using GeneMANIA webserver. Here genes are represented as nodes and edges indicate different types of interaction between genes. Black circles are the query genes and the color coding on edges indicate different types of interaction—which is defined in the network legend.

**Fig 7:**
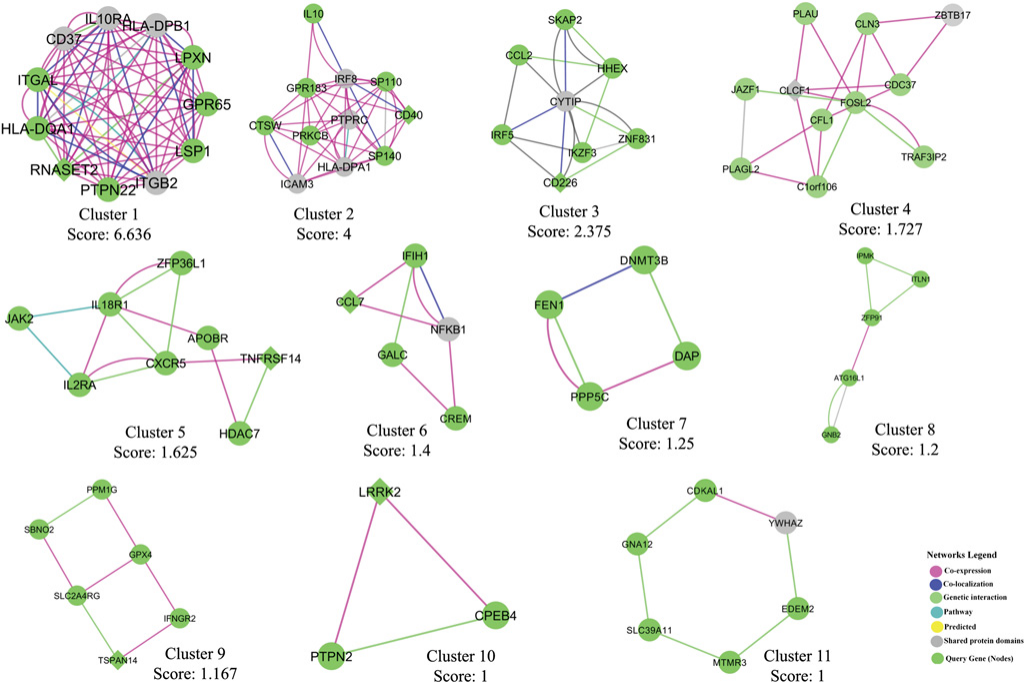
Top MCODE clusters from the gene interaction network. Highly connected gene clusters were identified from the network using MCODE v1.32 (a Cytoscape 2.8.2 plugin) and the top 11 clusters are presented (cutoff score ≥1).

We investigated the consensus between the results from the three classifiers, e.g. CADD, GWAVA and RegulomeDB. Here, we used a cutoff score for each classifier e.g. CADD ≥10, GWAVA ≥0.4 and RegulomeDB ≤2, to reduce false positive and to identify variants consistent in all three annotation schemes. A total of 929 SNPs (after removing the overlap) is found to be in agreement with any of the three classifiers’ cutoff score, and is plotted in the scatterplot (Fig. 5a), while the number of SNPs in concordance with different classifiers is depicted in the Venn diagram (Fig. 5b). It shows that eighteen SNPs (18 SNPs) from 15 GWAS loci are in concordance with the results of CADD, GWAVA and RegulomeDB (Table 2). However, we retrieved gene names for all the top ranked SNPs (total 929) using *Ensembl Variant Effect Predictor (VEP)* and constructed a gene interaction network from GeneMANIA webserver. And finally we identified highly connected gene clusters from the network using MCODE v1.32 (a Cytoscape 2.8.2 plugin). The gene interaction network is presented in Fig. 6 and top 11 MCODE gene clusters are shown in Fig. 7.

**Table 2:** List of prioritized putative regulatory SNPs.

Chromosome: Position	SNP	LD (*r2*)	Lead SNP	CADD	GWAVA	RegulomeDB	Genes ^¥^
**1:22706434**	rs6684375	1	rs12568930	13.02	0.44	2b	-
**2:198905874**	rs892513	0.97	rs1016883	14.41	0.4	1f	PLCL1
**5:55438580**	rs6859219	0.99	rs10065637	14.68	0.41	2b	ANKRD55
**6:90856878**	rs1010473	0.89	rs1847472	15.44	0.58	2b	BACH2
**6:138002175**	rs6927172	1	rs6920220	13.8	0.58	2b	-
**8:130604563**	rs13277237	0.89	rs1991866	12.78	0.58	2b	CCDC26
**10:35426755**	rs17499247	0.87	rs11010067	13.8	0.47	1f	CREM, RNU7-77P
**11:61564299**	rs4246215	1	rs4246215	10.67	0.78	1f	FADS1, FADS2, FEN1, MIR611
**11:65656564**	rs2231884	1	rs2231884	10.92	0.57	1b	CCDC85B, FIBP, FOSL1
**15:67442596**	rs17293632	1	rs17293632	13.47	0.46	2a	SMAD3
**16:23853860**	rs7200798	0.91	rs7404095	13.26	0.44	1f	PRKCB
**17:37912377**	rs12946510	1	rs12946510	11.41	0.5	1b	-
**17:37970149**	rs9909593	0.87	rs12946510	18.53	0.44	1f	IKZF3
**20:31349908**	rs6087990	0.82	rs4911259	13.54	0.68	2b	DNMT3B
**20:31380309**	rs1474738	0.99	rs4911259	13.11	0.42	2b	DNMT3B
**20:44735854**	rs6065926	0.99	rs1569723	16.82	0.65	1f	-
**20:44746982**	rs1883832	0.98	rs1569723	11.83	0.81	1f	CD40
**22:21980638**	rs5754426	0.85	rs2266959	10.1	0.42	2b	UBE2L3, YDJC

Position (hg19). Ref: Reference allele. Alt: Alternative allele. Cutoff: CADD ≥ 10, GWAVA ≥ 0.4 and RegulomeDB ≤ 2.

^¥^ Nearby genes are obtained using *Ensembl Variant Effect Predictor (VEP)* [39] and genes with HGNC [40] approved symbols are shown here.

### Evaluation of the prioritization scheme

We evaluated the above mentioned prioritization scheme in terms of fold-enrichment and strength of correlation (LD, *r* ^*2*^ ≥0.8) between the top 18 prioritized SNPs and lead GWAS SNPs from AS, CeD, IBD, Ps, RA and T1D (see Methods). The summary of the enrichment scores for genes of six IMDs along with the *p-values* (Fisher’s exact test) is provided in Table 3. We can see that all the enrichment scores for AS, CeD, IBD, IMD_5_, IMD_4_ and RA are statistically significant (*p-value* ≤0.01). Whereas for Ps, enrichment scores for CADD and GWAVA are statistically significant, and for T1D all enrichment scores but CADD+GWAVA+RegulomeDB are statistically significant. For IBD related genes, there is a gradual increase in the enrichment scores when more than one prediction tools are used for prioritization, while for other diseases more abrupt changes are observed (Table 3 and Figure A—Figure H in S1 File). It is clear from the comparison between IMD_4_ and IMD_5_, RA hugely contributes to the overall enrichment scores of IMD_5_; hence, we think it would be fair to compare the enrichment scores between IBD and IMD_4_. There is a gradual increase in the enrichment scores for IBD when more than one prediction tools are combined for SNP prioritization, while for IMD_4_ it decreases. However, the full list of genes for gene-enrichment analysis, and the summary of the gene overlap between the prediction tools and the reference diseases are available in S3 and S4 Tables, respectively.

**Table 3:** Summary of the enrichment scores for genes in six immune-mediated diseases (IMDs) using a combination of CADD, GWAVA and RegulomeDB prediction tool.

Reference Disease	Prediction Tool	Fold Enrichment	*P-value*
**Ankylosing spondylitis (AS)**	CADD	43.6	**7.13×10** ^**-10**^
	GWAVA	24.9	**2.94×10** ^**-6**^
	RegulomeDB	42.7	**1.27×10** ^**-8**^
	CADD+GWAVA	48.1	**2.09×10** ^**-6**^
	CADD+RegulomeDB	41	**1.19×10** ^**-3**^
	GWAVA+RegulomeDB	44.3	**2.92×10** ^**-6**^
	CADD+GWAVA+ RegulomeDB	69.8	**4.10×10** ^**-4**^
**Celiac disease (CeD)**	CADD	29.5	**3.95×10** ^**-13**^
	GWAVA	23.6	**4.64×10** ^**-12**^
	RegulomeDB	21.4	**6.24×10** ^**-8**^
	CADD+GWAVA	15.5	**1.05×10** ^**-3**^
	CADD+ RegulomeDB	26.5	**2.17×10** ^**-4**^
	GWAVA+ RegulomeDB	19	**6.88×10** ^**-5**^
	CADD+GWAVA+ RegulomeDB	45	**4.29×10** ^**-5**^
**Inflammatory bowel disease (IBD)**	CADD	55	**2.73×10** ^**-88**^
	GWAVA	51.2	**5.30×10** ^**-99**^
	RegulomeDB	55.7	**5.73×10** ^**-79**^
	CADD+GWAVA	52.2	**7.15×10** ^**-44**^
	CADD+ RegulomeDB	65.4	**8.85×10** ^**-36**^
	GWAVA+ RegulomeDB	57.7	**2.15×10** ^**-54**^
	CADD+GWAVA+ RegulomeDB	60.6	**1.34 ×10** ^**-19**^
**Psoriasis (Ps)**	CADD	13	**1.85×10** ^**-3**^
	GWAVA	17.3	**1.54×10** ^**-5**^
	RegulomeDB	9.9	0.02
	CADD+GWAVA	8.3	0.11
	CADD+ RegulomeDB	14.2	0.07
	GWAVA+ RegulomeDB	7.7	0.12
	CADD+GWAVA+ RegulomeDB	24.2	0.04
**Rheumatoid arthritis (RA)**	CADD	23.3	**4.30×10** ^**-13**^
	GWAVA	23.3	**6.04×10** ^**-16**^
	RegulomeDB	28.8	**2.81×10** ^**-15**^
	CADD+GWAVA	26.2	**1.29×10** ^**-8**^
	CADD+ RegulomeDB	51.2	**4.19×10** ^**-12**^
	GWAVA+ RegulomeDB	38	**1.51×10** ^**-14**^
	CADD+GWAVA+ RegulomeDB	76.1	**3.90×10** ^**-12**^
**Type 1 diabetes (T1D)**	CADD	26.8	**1.75×10** ^**-7**^
	GWAVA	35.7	**1.06×10** ^**-12**^
	RegulomeDB	35.7	**2.31×10** ^**-9**^
	CADD+GWAVA	25.9	**2.49×10** ^**-4**^
	CADD+ RegulomeDB	29.4	**2.25×10** ^**-3**^
	GWAVA+ RegulomeDB	47.6	**5.46×10** ^**-9**^
	CADD+GWAVA+ RegulomeDB	25	0.04
**IMD_5_**	CADD	21.8	**1.03×10** ^**-27**^
	GWAVA	20.7	**1.16×10** ^**-31**^
	RegulomeDB	21.3	**1.35×10** ^**-23**^
	CADD+GWAVA	17.2	**4.98×10** ^**-11**^
	CADD+RegulomeDB	26.7	**3.21×10** ^**-12**^
	GWAVA+RegulomeDB	24.5	**5.09×10** ^**-19**^
	CADD+GWAVA+RegulomeDB	36.3	**2.73×10** ^**-11**^
**IMD_4_**	CADD	22.3	**8.21×10** ^**-20**^
	GWAVA	22.6	**1.22×10** ^**-24**^
	RegulomeDB	20.1	**2.63×10** ^**-15**^
	CADD+GWAVA	13.6	**6.03×10** ^**-6**^
	CADD+ RegulomeDB	15.5	**1.34×10** ^**-4**^
	GWAVA+ RegulomeDB	20.9	**7.08×10** ^**-11**^
	CADD+GWAVA+ RegulomeDB	19.7	**4.65×10** ^**-4**^

Fold-enrichment was calculated as ((m/n)/(M/N)), where m is the number of prioritized genes (using a tool or a combination of tools) belong to the disease of interest, n is the total number of genes prioritized using a tool (or combination of tools), M is the total number of genes belong to the disease of interest, and N is the human genome background (30,000 genes). The *p-values* were obtained using Fisher’s exact test. Here, *p-values* less than or equal to 0.01 (≤0.01) are considered to be statistically significant (**bold**). For the enrichment analysis, reported genes (with HGNC approved gene symbol) were obtained from genome-wide studies of six immune-mediated diseases (IMDs): AS [16, 41], CeD [16], IBD [1], Ps [16, 42, 43], RA [16] and T1D [16]. *Ensembl Variant Effect Predictor (VEP)* [39] is used to obtain genes nearby the prioritized SNPs (only HGNC approved gene symbols are counted for the test). Full list of genes and the summary of the gene overlap are available in S3 and S4 Tables. **IMD**_**5**_: AS+CeD+Ps+RA+T1D; **IMD**_**4**_: AS+CeD+Ps+T1D.

Two SNPs are considered to be in strong LD, if the *r* ^*2*^ value between the two is greater than or equal to 0.8 (*r* ^*2*^ ≥0.8). We finally analyzed the strength of linkage-disequilibrium (LD) between the top 18 prioritized SNPs and lead GWAS SNPs of AS, CeD, IBD, Ps, RA and T1D (Table 4) for EUR population of the 1K Genomes Project. All the top 18 SNPs are in strong LD with the 15 GWAS SNPs of inflammatory bowel disease; while none of T1D associated GWAS SNPs had strong correlation. Rheumatoid arthritis associated seven GWAS SNPs are in strong LD with five of the prioritized SNPs. There are two celiac disease associated GWAS SNPs and one SNP each for ankylosing spondylitis and psoriasis in strong LD with the top prioritized SNPs.

**Table 4:** Summary of the linkage-disequilibrium (LD) (*r* ^*2*^ ≥0.8) between prioritized SNPs and lead SNPs from six immune-mediated diseases (IMDs).

Prioritized SNP	Ankylosing spondylitis	Celiac disease	Inflammatory bowel disease	Psoriasis	Rheumatoid arthritis	Type 1 diabetes
	Lead SNPs (*r* ^*2*^ ≥0.8)					
**rs6684375**	-	-	rs12568930 (1)	-	-	-
**rs892513**	-	-	rs1016883 (0.97)	-	-	-
**rs6859219**	-	-	rs10065637 (0.99)	-	rs6859219 (1)	-
**rs1010473**	-	-	rs1847472 (0.89)	-	-	-
**rs6927172**	-	rs2327832 (0.93)	rs6920220 (1)	-	rs6920220 (1)	-
**rs13277237**	-	-	rs1991866 (0.89)	-	-	-
**rs17499247**	-	-	rs11010067 (0.87)	-	-	-
**rs4246215**	-	-	rs4246215 (1)	-	-	-
**rs2231884**	-	-	rs2231884 (1)	-	-	-
**rs17293632**	-	-	rs17293632 (1)	-	-	-
**rs7200798**	-	-	rs7404095 (0.91)	-	-	-
**rs12946510**	-	-	rs12946510 (1)	-	-	-
**rs9909593**	-	-	rs12946510 (0.87)	-	-	-
**rs6087990**	-	-	rs4911259 (0.82)	-	-	-
**rs1474738**	-	-	rs4911259 (0.99)	-	-	-
**rs6065926**	-	-	rs1569723 (0.99)	-	rs4810485 (0.97); rs4239702 (0.85)	-
**rs1883832**	-	-	rs1569723 (0.98)	-	rs4810485 (1); rs4239702 (0.88)	-
**rs5754426**	rs2283790 (0.99)	rs2298428 (0.89)	rs2266959 (0.85)	rs4821124 (1)	rs11089637 (0.83)	**-**

The strength of correlation between the top 18 prioritized SNPs and lead SNPs from six IMDs e.g. AS [16, 41], CeD [16], IBD [1], Ps [16, 42, 43], RA [16] and T1D [16] were checked for European population of 1K Genomes Project. The lead SNPs for the six IMDs were retrieved from NHGRI GWAS catalog (and literatures), and the variants in strong linkage-disequilibrium (LD) (*r* ^*2*^ ≥0.8) with the lead GWAS SNPs were obtained using the HaploReg v2 webserver [26]. The LD variants’ list for each reference disease is manually screened for identifying the overlap with the top 18 prioritized SNPs.

## Discussion

The list of IBD associated GWAS loci is expanding very rapidly, which necessitates further investigation to pinpoint risk variants from associated loci and to elucidate the biological relevance of these associations. The task is not straightforward since most of the associated variants are noncoding, and interpreting the role of noncoding variants in disease pathogenesis is more challenging than coding variants—which have a direct effect on protein structure and function. However, noncoding variants are usually enriched in gene regulatory regions [46], and therefore annotations characteristic for regulatory sites, such as sequence conservation, chromatin state, expression quantitative trait loci (eQTL), transcription factor (TF) binding sites, etc. could be assigned to overlapping noncoding variants. Thus, variants with various regulatory annotations could be predicted functional for the underlying trait. So far, many studies being conducted to identify regulatory elements in human, where ENCODE project [32] is the biggest of its kind. Based on ENCODE data as well as other independent studies, many bioinformatics tools have been developed for annotating noncoding variants. In this study, we used four of these publicly available annotation tools, e.g. dbPSHP, CADD, GWAVA and RegulomeDB, for prioritizing IBD associated noncoding variants with potential gene regulatory functions.

We analyzed selective constraints on the 152 lead noncoding SNPs (representing 152 GWAS loci), since regulatory regions (e.g. transcription start sites (TSSs) and promoters) often overlap evolutionarily conserved sites in mammals [47], and therefore the functional importance of these SNPs can be explored in terms of constrained elements. The intensity of evolutionary constraint on each aligned position can be measured in terms of GERP++ rejected substitution (RS) [28]—where negative RS scores indicate regions under positive selection and positive RS scores indicate regions under selective constraint, i.e. negatively selected—which often correlates biological function [27]. It is apparent from GERP++ scores, ~43% (65 SNPs) IBD associated noncoding lead SNPs lie in evolutionary conserved regions (RS ≥0), and ~11% (16 SNPs) of these are under strong negative selection (RS ≥2). However, coding sequences are usually more conserved evolutionarily than noncoding regions of the genome. Therefore, analysis of population specific variations in regulatory regions, where evolutionary remodeling may extensively operate [47], is of greater importance for understanding human specific selection in the region [48]. Population specific neutrality estimates, e.g. Tajima’s D (TD) provided new insight about the selective constraints on IBD loci in European population. We demonstrated that around 30% (45 SNPs) of the noncoding variants are under strong balancing selection in CEU population (TD score >2) even though these regions are positively selected (GERP++ RS <0) in mammalian evolution (Fig. 1). We also identified 10 SNPs under strong selective constraint based on high GERP++ RS and TD scores (both RS & TD scores >2). These SNPs could contribute to IBD pathogenesis by disrupting gene expression. From cross population diversity estimates we can see more than 60% of the SNPs have higher derived allele frequency (ΔDAF >0) in CEU population than the rest of the world. This is understandable because the GWAS and meta-analysis data considered in this study was for people of European ancestry—which may also explain the high prevalence of IBD in this population.

However, not all GWAS identified SNPs are functional in disease pathogenesis—some variants may contribute to the disease directly and others through the variants in strong linkage-disequilibrium (LD) [49, 50]. Therefore, we investigated 152 lead noncoding SNPs along with 4,582 LD variants (total 4,734 variants) using three integrative annotation tools, e.g. CADD, GWAVA, and RegulomeDB, and identified 323 SNPs, 509 SNPs and 297 SNPs, respectively (total 929 SNPs, after removing overlap). Some of these 929 SNPs had very high annotation scores, some variants overlap gene regulatory regions, such as promoter, transcription factor binding sites, DNase I hypersensitive sites, and eQTLs, while few others had regulatory annotations from HGMD-PUBLIC. These variants are more likely to be functional in IBD. We presented gene interaction network and showed highly connected gene clusters—which represent different types of interaction between the prioritized genes (genes in the vicinity of the prioritized variants). In addition, we showed that IBD associated lead noncoding GWAS SNPs had higher CADD and GWAVA scores than the LD variants, which is consistent with previous findings [24, 25]. Similarly, a higher percent of lead GWAS SNPs had RegulomeDB scores <3 compared to the LD variants, which is comparable to a recent study where ~6.8% GWAS SNPs (3 out of 44 SNPs) had RegulomeDB score <3 against ~ 5.4% LD variants (31 out of 570 SNPs) [49].

We then analyzed the consensus between the three classifiers’ prioritized variants and found 68 SNPs common between CADD and GWAVA, 15 SNPs between CADD and RegulomeDB, and 81 SNPs between GWAVA and RegulomeDB. Among which rs2298428 (CADD = 24.5 & GWAVA = 0.61) and rs713875 (GWAVA = 0.47, RegulomeDB = 1f) are worth mentioning. SNP rs2298428 is found to be associated with IBD and celiac disease [51], and is also a cis-eQTL associated with IBD [52, 53], while SNP rs713875 is a cis-regulated ASM (allele-specific DNA methylation) variant associated with IBD [54].

Finally, through concordance analysis we identified 18 SNPs from 15 IBD associated GWAS loci. These noncoding variants are more likely to have regulatory functions and may contribute to IBD pathogenesis by dysregulating the expression of nearby genes. Four of these eighteen prioritized SNPs are lead GWAS SNPs, which indicates that some of the lead GWAS SNPs are true causal variants for the underlying trait, while the effect of the remaining variants could be inferred from the variants in strong LD with the lead GWAS SNPs. Interestingly, some of these 18 SNPs and genes in the vicinity are found to be associated with cancer and other immune-mediated diseases (IMDs). For example, rs5754426 is the top ranked variant among 92 SNPs in the locus—which includes some newly identified SNPs associated with celiac disease (*UBE2L3* rs2298428) [55], Crohn’s disease (CD) and psoriasis (*YDJC* rs181359) [56], as well as chronic hepatitis B virus infection (*UBE2L3* rs4821116) [57]. Another top ranked SNP rs1883832 (*CD40*) is found to be associated with atherosclerosis in Chinese population [58]. Moreover, a recent study in Russians showed the association of two *CD40* variants rs6074022 and rs1883832 with multiple sclerosis (MS)—where only rs6074022 reached genome-wide significance [59]. In contrast, our results suggest that rs1883832 and rs6065926 are the top two putative regulatory variants from the locus, where the former has known regulatory function in *CD40* mRNA translation [60]. Besides, it is found that the loci rs2231884 (*FOSL1*, *FIBP*, *CCDC85B*) and rs2266959 (*UBE2L3*, *YDJC*) overlap active DNA regulatory elements (DRE) of the intestine and immune cells of IBD patients, while rs17293632 (*SMAD3*), rs7404095 (*PRKCB*) and rs12946510 only overlap DRE of immune cells [61]. However, there are evidences for other prioritized SNPs as well, such as, rs6859219 (*ANKRD55*) is found to be associated with MS risk [62]; rs6927172 (*TNFAIP3*) is found to be associated with anti-TNF therapy response among IBD patients [63]; rs4246215 (*FEN1*) is associated with DNA damage [64] and increased risk of colorectal cancer [65]; and rs17293632 (*SMAD3*) is found to be associated with the recurrence of surgery in CD patients [66]. These indicate the quality of CADD, GWAVA, and RegulomeDB findings, and show the functional relevance of the prioritized variants.

Furthermore, our analysis showed that a combination of these three tools could be very effective to annotate and prioritize putative functional noncoding variants from GWAS loci. To evaluate this, we analyzed the enrichment scores for genes of IBD and five other IMDs (e.g. AS, CeD, Ps, RA and T1D), as well as strength of linkage-disequilibrium (LD) between the top prioritized SNPs and lead GWAS SNPs from those diseases. We observed rapid changes in the enrichment scores at each level of prioritization, i.e. variants prioritized by any one tool, any two tools, or a combination of all three (Table 3). And it is apparent from the analysis, for IBD there is a consistent increase in the enrichment scores when multiple programs are used, while for IMD_4_ (combined genes from AS, CeD, Ps and T1D) there is a decrease in the enrichment. It is noticeable, for IBD the enrichment was highest (~65 fold) when CADD+RegulomeDB is used compared to any other combination of tools—which may be due to the relatively higher number of genes containing loci; nonetheless, the final enrichment (CADD+GWAVA+RegulomeDB, ~61 fold) is also considerable. Moreover, the top 18 SNPs are in strong linkage-disequilibrium (LD) (*r* ^*2*^ ≥0.8) with IBD associated 15 lead GWAS SNPs (Table 4). These results indicate the relatedness of the proposed model to prioritize regulatory variants from the GWAS loci.

An interesting observation was the significant level of enrichment for other IMDs, which could be partly explained in terms of pleiotropy—a phenomenon of shared loci where variants in the same gene could lead to different phenotypic effects. Pleiotropy is very common in complex traits, such as immune-mediated diseases (IMDs) [18]. For example, in IBD 70% (113 out of 163) of the loci are shared with other diseases or traits, and around 40% (66 out of 163) of this overlap is with IMDs [1]. However, our studied loci had 29% (44 out of 152) overlap with IMD_5_, which eventually reached to 40% (6 out of 15) in the final level of prioritization; this indicates the functional importance of these loci in autoimmune diseases in general. Nonetheless, the direction of this sharing is not same for all the overlapping loci; some are concordant, while others are discordant. For example, the variants of rs10065637, rs6920220, rs12946510, and rs2266959 loci are associated with increased risk of IBD and RA (i.e. same effect), but rs1569723 variants have opposite effect (discordant) on the phenotypes (for detail analysis ref. [18]).

Although, we were successful in identifying few variants with probable regulatory functions from the studied GWAS loci (which was the main aim of this study), we could not be conclusive for their association with IBD or other IMDs, due to the limitation of our *in-silico* method (none of the tools were disease specific; regulatory elements (e.g. TF binding sites, eQTLs) mostly for certain cell types from ENCODE). However, some of the results are well aligned with other literary evidences from IBD patients, where we could suspect their involvement with the disease. Overall, with a combination of constrained elements and prediction tools we were able to cover around 95% (145 out of 152) of the loci, though we cannot infer the functional importance of the remaining loci using our methods.

This study showed a method of post-GWAS data analysis leveraging the publicly available bioinformatics resources. We found integrative annotation a very powerful tool for prioritizing functional variants from the background, which could be adopted in the upstream or downstream analysis to optimize GWAS findings. However, in spite of the compelling evidences, the findings of this computational prediction should be handled with caution and must be verified experimentally using appropriate systems before considering for genomic medicine.

## Supporting Information

S1 Table Summary of GERP++, TD, DAF and ΔDAF scores for the 152 lead noncoding GWAS SNPs. (DOCX)

S2 Table Summary of integrative annotation scores of IBD associated lead noncoding SNPs and variants in strong linkage-disequilibrium (LD) with the lead SNPs, using CADD, GWAVA and RegulomeDB tools. (XLSX)

S3 Table Full list of genes used in gene-enrichment analysis. (PDF)

S4 Table Summary of the gene overlap between the reference disease and the prediction tool. (PDF)

S1 File Enrichment scores for AS, CeD, IBD, Ps, RA, T1D, IMD4 and IMD5 are presented in line-graphs in Figure A—Figure H in S1 File, respectively. (PDF)

S2 File Gene interaction network (Cytoscape Session). (RAR)
