# Supplementary material for: In-Silico Analysis of Inflammatory Bowel Disease (IBD) GWAS Loci to Novel Connections
Source: PLoS One. 2015 Mar 18;10(3):e0119420. doi: 10.1371/journal.pone.0119420 (PMC4364731; doi:10.1371/journal.pone.0119420)
Supplement: S1 Table — (DOCX) [file pone.0119420.s001.docx]

S1 Table. Summary of GERP++, TD, DAF and ΔDAF scores for the 152 lead noncoding GWAS SNPs.

|  |  |  |  |  |  |  |  |  |  |
| --- | --- | --- | --- | --- | --- | --- | --- | --- | --- |
| SNP | Chromosome | Position | Ref | Alt | GERP++ RS | GERP++ NR | TD | ΔDAF | DAF |
| rs4656958 | 1 | 160856964 | A | G | 0.45 | 2.41 | 3.58 | 0.0241 | 0.3412 |
| rs10797432 | 1 | 2501338 | C | T | -1.22 | 0.61 | 2.61 | -0.0650 | 0.4529 |
| rs4845604 | 1 | 151801680 | G | A | -7.55 | 3.77 | 1.89 | -0.0189 | 0.1412 |
| rs670523 | 1 | 155878732 | A | G | 3.75 | 3.75 | 1.84 | 0.3136 | 0.6706 |
| rs3897478 | 1 | 120451190 | T | C | 0.10 | 2.61 | -0.80 | -0.0756 | 0.0824 |
| rs2816958 | 1 | 200101920 | A | G | -6.07 | 3.03 | 1.92 | 0.0495 | 0.9176 |
| rs12568930 | 1 | 22702231 | T | C | 0.48 | 2.91 | 1.18 | -0.0178 | 0.1941 |
| rs17391694 | 1 | 78623626 | C | T | -6.18 | 3.09 | 1.93 | 0.1064 | 0.1471 |
| rs7554511 | 1 | 200877562 | C | A | 3.45 | 4.36 | 2.55 | 0.1620 | 0.3176 |
| rs6679677 | 1 | 114303808 | C | A | -0.34 | 3.66 | 0.93 | 0.0739 | 0.1118 |
| rs2651244 | 1 | 70995562 | G | A | -0.26 | 0.92 | 1.49 | 0.1644 | 0.4059 |
| rs2488389 | 1 | 197631141 | G | A | -5.20 | 2.77 | 1.59 | -0.0459 | 0.1882 |
| rs3024505 | 1 | 206939904 | G | A | -5.96 | 2.98 | 1.22 | 0.0973 | 0.1882 |
| rs9286879 | 1 | 172862234 | A | G | 2.92 | 4.09 | 2.26 | -0.2513 | 0.2176 |
| rs35675666 | 1 | 8021973 | G | T | -0.33 | 2.85 | 1.46 | 0.0207 | 0.1765 |
| rs6426833 | 1 | 20171860 | G | A | 1.51 | 3.82 | 2.46 | -0.0037 | 0.5353 |
| rs1517352 | 2 | 191931464 | A | C | 0.08 | 2.94 | 0.56 | 0.2214 | 0.6529 |
| rs2382817 | 2 | 219151218 | A | C | -1.00 | 2.96 | 3.57 | 0.0645 | 0.6765 |
| rs6740462 | 2 | 65667272 | C | A | 0.37 | 2.91 | 2.39 | 0.0798 | 0.2765 |
| rs7608910 | 2 | 61204856 | A | G | 1.63 | 2.97 | 2.53 | 0.0904 | 0.3824 |
| rs1016883 | 2 | 198881668 | G | A | -3.92 | 4.24 | 3.23 | -0.0691 | 0.1412 |
| rs2111485 | 2 | 163110536 | A | G | -0.78 | 2.55 | -1.47 | 0.2372 | 0.6059 |
| rs10865331 | 2 | 62551472 | A | G | -0.45 | 0.23 | 3.12 | -0.1555 | 0.2824 |
| rs6545800 | 2 | 25118885 | C | T | 0.50 | 0.50 | 2.64 | 0.0871 | 0.5765 |
| rs6716753 | 2 | 231097129 | T | C | 1.65 | 1.65 | 1.65 | -0.0802 | 0.7882 |
| rs10495903 | 2 | 43806918 | C | T | -5.64 | 2.82 | 3.15 | 0.0658 | 0.1588 |
| rs12994997 | 2 | 234173503 | G | A | -6.12 | 3.94 | 3.01 | 0.1848 | 0.5706 |
| rs917997 | 2 | 103070568 | T | C | -1.74 | 2.57 | 3.31 | -0.0684 | 0.2059 |
| rs925255 | 2 | 28614794 | C | T | 1.71 | 4.72 | 2.27 | 0.1189 | 0.4412 |
| rs17229285 | 2 | 199523122 | C | T | 5.77 | 5.77 | 3.11 | 0.2624 | 0.5882 |
| rs1728918 | 2 | 27635463 | A | G | -1.82 | 1.13 | 2.99 | 0.0358 | 0.2647 |
| rs9847710 | 3 | 53062661 | T | C | 0.47 | 0.47 | 1.90 | 0.0072 | 0.5176 |
| rs4256159 | 3 | 18767404 | C | T | 4.88 | 5.77 | 2.58 | 0.0550 | 0.1706 |
| rs2472649 | 4 | 74857708 | A | G | -0.20 | 2.26 | 0.31 | 0.1938 | 0.8353 |
| rs13126505 | 4 | 102865304 | G | A | -2.54 | 2.72 | 0.81 | 0.0314 | 0.0588 |
| rs3774959 | 4 | 103511114 | G | A | 3.16 | 3.16 | 1.64 | -0.0178 | 0.3588 |
| rs7657746 | 4 | 123161619 | A | G | 1.25 | 5.20 | 0.48 | -0.0278 | 0.1824 |
| rs6837335 | 4 | 48363983 | A | G | -1.72 | 1.48 | 2.83 | 0.1304 | 0.6647 |
| rs11739663 | 5 | 594083 | T | C | -1.66 | 0.83 | 2.01 | -0.0131 | 0.7588 |
| rs2188962 | 5 | 131770805 | C | T | -0.56 | 3.56 | 3.71 | 0.1939 | 0.3824 |
| rs2930047 | 5 | 10695526 | T | C | -0.29 | 3.72 | 1.03 | 0.1675 | 0.5882 |
| rs7702331 | 5 | 72551134 | A | G | -1.54 | 4.71 | 3.20 | -0.0204 | 0.6118 |
| rs254560 | 5 | 134443606 | G | A | 1.71 | 2.59 | 2.47 | 0.0935 | 0.3824 |
| rs1363907 | 5 | 96252803 | G | A | -2.35 | 1.18 | 1.69 | 0.1225 | 0.4647 |
| rs6863411 | 5 | 141513204 | A | T | 1.04 | 3.38 | 2.51 | -0.0573 | 0.6118 |
| rs6871626 | 5 | 158826792 | C | A | -3.44 | 3.08 | 2.31 | 0.0230 | 0.3294 |
| rs17695092 | 5 | 173337853 | T | G | -3.77 | 4.52 | 2.49 | -0.1227 | 0.7235 |
| rs11741861 | 5 | 150277909 | A | G | -0.88 | 3.51 | -0.77 | -0.1071 | 0.0588 |
| rs4836519 | 5 | 130017287 | T | C | 0.00 | 0.00 | 0.03 | 0.1069 | 0.2824 |
| rs10065637 | 5 | 55438851 | C | T | 1.65 | 4.46 | 1.73 | 0.0933 | 0.2118 |
| rs11742570 | 5 | 40410584 | T | C | -3.35 | 3.91 | 3.30 | -0.1479 | 0.3765 |
| rs12654812 | 5 | 176794191 | G | A | 1.63 | 2.51 | 1.70 | 0.0448 | 0.3588 |
| rs9491697 | 6 | 127456122 | A | G | -0.65 | 3.37 | 3.37 | 0.0045 | 0.4235 |
| rs3851228 | 6 | 111848191 | A | T | 1.92 | 1.92 | 1.20 | -0.0483 | 0.0353 |
| rs6920220 | 6 | 138006504 | G | A | 2.71 | 3.58 | 2.26 | 0.0377 | 0.1529 |
| rs1847472 | 6 | 90973159 | C | A | 2.42 | 4.22 | 1.68 | 0.1375 | 0.3471 |
| rs12199775 | 6 | 143898894 | A | G | 0.92 | 3.47 | 4.04 | 0.0375 | 0.0824 |
| rs9358372 | 6 | 20812588 | G | A | 0.57 | 1.47 | 2.58 | 0.0635 | 0.6588 |
| rs17119 | 6 | 14719496 | G | A | 0.09 | 2.96 | 1.91 | 0.0499 | 0.7706 |
| rs12663356 | 6 | 21430728 | T | C | -3.99 | 3.64 | 3.57 | 0.1002 | 0.5824 |
| rs9264942 | 6 | 31274380 | T | C | 0.61 | 1.51 | NA | NA | NA |
| rs212388 | 6 | 159490436 | C | T | -0.87 | 0.44 | 1.03 | 0.0992 | 0.5471 |
| rs1819333 | 6 | 167373547 | T | G | 0.17 | 1.39 | 3.42 | -0.0718 | 0.5294 |
| rs6568421 | 6 | 106435025 | A | G | -0.05 | 4.35 | 2.17 | 0.1472 | 0.3176 |
| rs6927022 | 6 | 32612397 | A | G | -2.03 | 2.14 | NA | NA | NA |
| rs13204742 | 6 | 128245765 | G | T | 1.29 | 1.29 | 2.09 | 0.1058 | 0.1588 |
| rs1734907 | 7 | 100315517 | A | G | -0.43 | 1.55 | 2.49 | 0.0438 | 0.1765 |
| rs38904 | 7 | 116892846 | T | C | -0.72 | 0.36 | 1.90 | 0.0239 | 0.5235 |
| rs864745 | 7 | 28180556 | T | C | -8.42 | 4.21 | 1.53 | 0.1480 | 0.5118 |
| rs4380874 | 7 | 107480315 | T | C | -1.18 | 4.17 | 2.30 | 0.1302 | 0.4176 |
| rs9297145 | 7 | 98759117 | C | A | -0.24 | 1.71 | 1.71 | -0.0144 | 0.6882 |
| rs798502 | 7 | 2789880 | A | C | -1.06 | 0.63 | 1.30 | 0.0308 | 0.2706 |
| rs4728142 | 7 | 128573967 | G | A | -0.08 | 2.82 | 2.75 | 0.0851 | 0.3941 |
| rs10486483 | 7 | 26892440 | G | A | 5.04 | 5.04 | 2.78 | -0.0430 | 0.7706 |
| rs4722672 | 7 | 27231762 | C | T | 2.99 | 4.79 | -1.06 | 0.1576 | 0.8353 |
| rs1456896 | 7 | 50304461 | C | T | -2.38 | 2.58 | 2.23 | -0.0132 | 0.3706 |
| rs1991866 | 8 | 130624105 | G | C | -0.01 | 1.96 | 2.09 | 0.0258 | 0.4706 |
| rs921720 | 8 | 126534671 | A | G | -5.13 | 3.43 | 2.76 | 0.1533 | 0.6059 |
| rs7015630 | 8 | 90875918 | T | C | 0.15 | 0.15 | 3.09 | -0.0572 | 0.2353 |
| rs6651252 | 8 | 129567181 | T | C | 3.36 | 5.74 | 0.31 | -0.0551 | 0.1000 |
| rs10758669 | 9 | 4981602 | C | A | 0.38 | 2.87 | 0.07 | 0.0528 | 0.3647 |
| rs4743820 | 9 | 93928416 | C | T | -1.40 | 1.35 | 2.16 | 0.1201 | 0.7412 |
| rs4246905 | 9 | 117553249 | T | C | -4.15 | 3.99 | 1.27 | 0.0000 | 1.0000 |
| rs2227564 | 10 | 75673101 | T | C | 3.07 | 5.47 | 0.94 | 0.0538 | 0.2529 |
| rs2790216 | 10 | 59997926 | G | A | -2.79 | 3.82 | 1.99 | -0.1588 | 0.2118 |
| rs7911264 | 10 | 94436851 | T | C | 1.79 | 1.79 | 1.23 | 0.0090 | 0.4412 |
| rs6586030 | 10 | 82254047 | A | G | -0.43 | 3.07 | 2.99 | 0.1070 | 0.1824 |
| rs10761659 | 10 | 64445564 | A | G | -0.69 | 4.31 | 1.39 | 0.0516 | 0.5353 |
| rs12722515 | 10 | 6081230 | C | A | 0.69 | 0.69 | 1.83 | 0.0333 | 0.1353 |
| rs1250546 | 10 | 81032532 | A | G | -1.48 | 2.61 | 2.68 | -0.0812 | 0.5765 |
| rs4409764 | 10 | 101284237 | T | G | 1.22 | 4.97 | 4.21 | 0.0266 | 0.5294 |
| rs11010067 | 10 | 35295431 | C | G | -1.71 | 2.17 | 1.81 | 0.0220 | 0.6412 |
| rs559928 | 11 | 64150370 | T | C | -1.07 | 0.53 | 2.10 | -0.0096 | 0.8118 |
| rs2155219 | 11 | 76299194 | G | T | -2.08 | 2.30 | -0.34 | 0.0387 | 0.5294 |
| rs10896794 | 11 | 58339124 | T | C | -1.68 | 3.50 | 1.82 | 0.0308 | 0.2353 |
| rs483905 | 11 | 96023427 | G | A | 2.28 | 4.38 | 2.03 | 0.0625 | 0.3235 |
| rs561722 | 11 | 114386830 | C | T | -0.48 | 1.71 | 1.88 | -0.1454 | 0.2588 |
| rs6592362 | 11 | 87125438 | A | G | 0.00 | 0.00 | 1.50 | -0.2152 | 0.2118 |
| rs4246215 | 11 | 61564299 | G | T | 0.22 | 3.83 | 1.85 | 0.0302 | 0.3824 |
| rs907611 | 11 | 1874072 | G | A | -1.50 | 2.43 | 3.13 | 0.0674 | 0.3118 |
| rs630923 | 11 | 118754353 | C | A | 1.38 | 4.52 | 1.48 | 0.0213 | 0.1353 |
| rs2231884 | 11 | 65656564 | C | T | -3.09 | 2.79 | 2.18 | 0.0209 | 0.2176 |
| rs7134599 | 12 | 68500075 | G | A | -0.77 | 3.36 | 1.45 | 0.1886 | 0.4059 |
| rs11168249 | 12 | 48208368 | T | C | 1.17 | 3.00 | 1.80 | -0.1009 | 0.5118 |
| rs11612508 | 12 | 12657513 | A | G | -0.25 | 2.37 | 3.27 | 0.1066 | 0.2824 |
| rs11564258 | 12 | 40792300 | G | A | -3.97 | 3.38 | 0.21 | 0.0311 | 0.9765 |
| rs941823 | 13 | 41013977 | T | C | -1.20 | 2.07 | 1.72 | -0.0125 | 0.7706 |
| rs9557195 | 13 | 99956622 | T | C | -6.10 | 3.05 | 1.65 | 0.1269 | 0.2529 |
| rs17085007 | 13 | 27531267 | T | C | -2.41 | 3.42 | 2.58 | -0.0191 | 0.1412 |
| rs4899554 | 14 | 75701221 | C | T | -2.21 | 2.90 | 2.37 | 0.0060 | 0.1471 |
| rs8005161 | 14 | 88472595 | C | T | -8.39 | 4.19 | 2.70 | -0.0756 | 0.1235 |
| rs194749 | 14 | 69273905 | T | C | -7.52 | 3.76 | 2.91 | 0.0339 | 0.7765 |
| rs16967103 | 15 | 38899190 | T | C | 1.56 | 2.49 | 1.86 | -0.0916 | 0.7471 |
| rs7495132 | 15 | 91172901 | C | T | 0.89 | 2.94 | 0.66 | -0.0119 | 0.1059 |
| rs28374715 | 15 | 41563950 | A | G | -3.41 | 2.56 | NA | NA | NA |
| rs17293632 | 15 | 67442596 | C | T | 4.63 | 4.63 | 2.64 | 0.0703 | 0.1941 |
| rs7404095 | 16 | 23864590 | T | C | 1.43 | 3.40 | 2.40 | -0.0345 | 0.3765 |
| rs10521318 | 16 | 86011337 | C | T | -0.31 | 1.75 | 2.33 | 0.0354 | 0.0941 |
| rs1728785 | 16 | 68591230 | A | C | 1.48 | 2.45 | 1.71 | -0.0400 | 0.1471 |
| rs11150589 | 16 | 30482494 | T | C | 0.46 | 0.46 | 1.87 | -0.0350 | 0.5588 |
| rs26528 | 16 | 28517709 | T | C | -3.51 | 3.82 | 2.54 | -0.0699 | 0.5577 |
| rs529866 | 16 | 11373320 | C | T | 0.02 | 1.11 | 2.52 | 0.0409 | 0.1647 |
| rs3091316 | 17 | 32593974 | G | A | -0.74 | 0.39 | 1.85 | -0.0849 | 0.3235 |
| rs12942547 | 17 | 40527544 | A | G | -1.88 | 0.94 | 3.32 | -0.0538 | 0.5588 |
| rs2945412 | 17 | 25843643 | G | A | 2.67 | 2.67 | 2.27 | 0.0029 | 0.4118 |
| rs12946510 | 17 | 37912377 | C | T | 0.90 | 4.32 | 0.84 | 0.1316 | 0.4824 |
| rs7210086 | 17 | 70641698 | A | C | -4.20 | 3.36 | 2.38 | 0.0369 | 0.2000 |
| rs1893217 | 18 | 12809340 | A | G | -0.58 | 2.06 | 1.13 | 0.0085 | 0.1235 |
| rs727088 | 18 | 67530439 | G | A | 0.39 | 2.95 | 2.72 | 0.0106 | 0.4882 |
| rs7240004 | 18 | 46395022 | A | G | 1.61 | 1.61 | 1.78 | 0.1371 | 0.6765 |
| rs11672983 | 19 | 55383051 | G | A | 0.56 | 0.56 | 2.11 | 0.0493 | 0.3706 |
| rs516246 | 19 | 49206172 | C | T | -4.82 | 2.41 | 4.31 | 0.1705 | 0.4941 |
| rs2024092 | 19 | 1124031 | G | A | -6.25 | 3.13 | 2.08 | -0.0261 | 0.2118 |
| rs1126510 | 19 | 47123783 | A | G | 0.85 | 3.18 | 2.03 | 0.0963 | 0.3412 |
| rs4802307 | 19 | 46849806 | G | T | -1.85 | 1.76 | 0.13 | 0.0946 | 0.2706 |
| rs17694108 | 19 | 33731551 | G | A | -2.85 | 2.28 | 1.64 | 0.0864 | 0.3176 |
| rs11879191 | 19 | 10512911 | G | A | -1.23 | 1.81 | 2.14 | -0.0353 | 0.1941 |
| rs6017342 | 20 | 43065028 | A | C | 0.23 | 0.23 | 2.21 | 0.1715 | 0.4647 |
| rs4911259 | 20 | 31376282 | T | G | 0.76 | 0.76 | 2.93 | -0.1932 | 0.3529 |
| rs6142618 | 20 | 30725648 | A | G | -2.78 | 2.43 | NA | NA | NA |
| rs6062504 | 20 | 62348907 | A | G | 0.00 | 0.00 | 2.42 | 0.0115 | 0.6529 |
| rs913678 | 20 | 48955424 | T | C | 3.06 | 4.09 | 2.11 | 0.2338 | 0.6118 |
| rs1569723 | 20 | 44742064 | C | A | 0.84 | 3.22 | 1.57 | -0.0076 | 0.7588 |
| rs6088765 | 20 | 33799280 | T | G | 1.38 | 2.33 | 1.48 | 0.0892 | 0.5941 |
| rs259964 | 20 | 57824309 | A | G | -4.92 | 2.46 | 1.96 | -0.0153 | 0.5706 |
| rs2284553 | 21 | 34776695 | A | G | -3.95 | 1.97 | 0.48 | 0.0000 | 1.0000 |
| rs2823286 | 21 | 16817938 | G | A | -2.77 | 4.08 | 3.36 | -0.0566 | 0.7059 |
| rs7282490 | 21 | 45615741 | G | A | -2.44 | 1.22 | 3.22 | 0.0461 | 0.6118 |
| rs2836878 | 21 | 40465534 | G | A | -2.51 | 1.28 | 3.88 | -0.1126 | 0.6706 |
| rs2266959 | 22 | 21922904 | G | T | -0.01 | 3.37 | 1.36 | -0.0704 | 0.1588 |
| rs2412970 | 22 | 30486826 | A | G | -0.78 | 0.39 | 0.99 | 0.1358 | 0.6294 |
| rs2413583 | 22 | 39659773 | C | T | -0.21 | 0.88 | 0.12 | -0.0052 | 0.8294 |

TD, DAF & ΔDAF are estimated for CEU population of 1K Genomes project.

NA: data not available. Chr: Chromosome. Pos: Position (1-based, hg19). Ref: Reference allele. Alt: Alternative allele. GERP++ RS: GERP++ rejected substitution. GERP++ NR: GERP++ neutral rate. TD: Tajima’s D. DAF: Derived allele frequency. ΔDAF: difference in DAF.
