# Supplementary material for: In-Silico Analysis of Inflammatory Bowel Disease (IBD) GWAS Loci to Novel Connections
Source: PLoS One. 2015 Mar 18;10(3):e0119420. doi: 10.1371/journal.pone.0119420 (PMC4364731; doi:10.1371/journal.pone.0119420)
Supplement: S3 Table — (PDF) [file pone.0119420.s003.pdf]

**S3 Table. Full list of genes used in gene-enrichment analysis.**

| Disease | Number of Genes | Genes |
|---|---|---|
| **Ankylosing spondylitis (AS)** | 43 | ANO6, ANTXR2, B3GNT2, BACH2, CARD9, EDIL3, EOMES, ERAP1, ERAP2, FCGR2A, GPR25, GPR35, GPR37, GPR65, HAPLN1, HHAT, HLA-A, HLA-B, ICOSLG, IL1R1, IL1R2, IL6R, IL7R, IL12B, IL23R, IL27, KIF21B, LTBR, NKX2-3, NOS2, NPEPPS, PSMG1, PTGER4, RUNX3, SH2B3, SULT1A1, TBKBP1, TBX21, TNFRSF1A, TYK2, UBE2E3, UBE2L3, ZMIZ1 |
| **Celiac disease (CeD)** | 100 | ADAD1, AHSA2, ANKFN1, ASCL2, ATXN2, BACH2, BAGE, BAGE2, C4orf17, CCR1, CCR2, CCR3, CCR4, CCR5, CCR9, CCRL2, CCT8L2, CD28, CD80, CD247, CIITA, CLEC16A, CTLA4, DOK5, EAPP, ELMO1, EMX2, EMX2OS, ETS1, FASLG, FRMD4B, H19, HLA-DQA1, HLA-DQB1, HYDIN, ICOS, ICOSLG, IGF2, IL1RL1, IL1RL2, IL2, IL12A, IL18R1, IL18RAP, IL21, INS, IRF4, ITGA4, KIAA1109, KLK2, KLK3, KLK4, KLK15, KLKP1, LPP, LSP1, MAP3K7, MMEL1, MRPL23, MTTP, NAV1, NFIA, NOG, PARK7, PLEK, PPP1R12B, PTPN2, PTPRK, RAB11FIP2, REL, RGS1, RUNX3, SCHIP1, SH2B3, SLC9A4, SNX6, SOCS1, STAC, STK32B, SYT2, SYT8, TAGAP, TH, THEMIS, TLR7, TLR8, TNFAIP3, TNFRSF9, TNFRSF14, TNFSF4, TNFSF18, TNNI2, TNNT3, TPTE, UBE2E3, UBE2L3, UBE2T, YDJC, ZFP36L1, ZMIZ1 |
| **Inflammatory bowel disease (IBD)** | 297 | ACSL6, ADA, ADAM30, ADCY3, ADCY7, AHSA2, APOBEC3G, ARHGAP30, ARPC2, ATF4, ATG16L1, BRE, BSN, C1orf53, C2orf74, CALM3, CARD9, CARD11, CCDC88B, CCDC116, CCL2, CCL11, CCL13, CCR6, CD5, CD6, CD40, CD48, CD226, CD244, CEBPB, CEBPG, CEP250, CISD1, CNTF, CPEB4, CREB5, CREM, CRTC3, CSF2, CTDSP1, CTSW, CTSZ, CXCL1, CXCL2, CXCL3, CXCL5, CXCL6, CXCR1, CXCR2, CXCR5, DAP, DBP, DCLRE1B, DLD, DNMT3B, DOK3, EIF3C, EPO, ERAP1, ERAP2, F11R, FADS1, FADS2, FAIM3, FASLG, FCGR2A, FCGR2B, FCGR3A, FCGR3B, FCRLA, FLRT1, FOS, FOSL1, FOSL2, FUT2, FYN, GALC, GART, GNA12, GPR18, GPR35, GPR65, GPR183, GPX1, GPX4, GSDMA, GSDMB, HCK, HLA-C, HLA-DQA1, HLA-DQB1, HLA-DRA, HLA-DRB1, HMHA1, HNF4A, HSPA6, ICAM1, ICOSLG, IFIH1, IFNAR1, IFNAR2, IFNG, IFNGR2, IKZF1, IKZF3, IL1R1, IL1R2, IL1RL1, IL1RL2, IL2, IL2RA, IL3, IL4, IL5, IL6ST, IL10, IL10RB, IL12B, IL12RB2, IL13, IL15RA, IL18R1, IL18RAP, IL19, IL20, IL21, IL22, IL23R, IL24, IL26, IL27, IL31RA, INPP5D, INPP5E, IP6K1, IP6K2, IPMK, IRF1, IRF5, IRF8, IRGM, ITGAL, |

| | | |
|---|---|---|
| | | ITIH4, ITLN1, ITPKA, IZUMO1, JAK2, JAZF1, JRKL, KIAA1841, KIF21B, KIR2DL1, LACC1, LGALS9, LIF, LILRB4, LIME1, LITAF, LNPEP, LOH12CR1, LPXN, LRRK2, LSP1, MAML2, MANBA, MAP3K8, MAPK1, MAPKAPK2, MICB, MLH3, MMEL1, MMP9, MST1, MST1R, MSTO1, MTMR3, MUC19, NDFIP1, NDUFAF1, NFIL3, NFKB1, NFKBIL1, NKX2-3, NLRP2, NLRP7, NOD2, NOS2, NUPR1, NUSAP1, NXPE1, NXPE4, ORMDL3, OSM, PDLIM4, PF4, PF4V1, PFKFB4, PHACTR2, PIGR, PLCH2, PLCL1, PLTP, PMPCA, PNKD, PPAN-P2RY11, PRKCB, PRKCD, PROCR, PSORS1C1, PTGDR2, PTGER4, PTPN22, RABEP2, RASGRP1, RASSF5, REL, RELA, REV3L, RFTN2, RIMBP3, RIPK2, RIT1, RMI2, RNASET2, RORC, RPS6KA2, RPS6KA4, RPS6KB1, SDCCAG3, SLAMF1, SLAMF7, SLC2A4RG, SLC9A3, SLC10A4, SLC11A1, SLC22A4, SLC22A5, SMAD3, SMAD7, SMURF1, SNX32, SOCS1, SP140, SPHK2, SPRED1, SPRED2, SPRY4, STAT1, STAT3, STAT4, STAT5A, STAT5B, SULT1A1, SULT1A2, TAB1, TAGAP, TEC, TMBIM1, TMEM50B, TNC, TNFAIP3, TNFRSF4, TNFRSF6B, TNFRSF9, TNFRSF14, TNFRSF18, TNFSF8, TNFSF15, TNFSF18, TNIP1, TNNI2, TNPO3, TRAF3IP2, TRIB1, TRPT1, TSPAN14, TSPAN33, TTYH3, TUBD1, TXK, TYK2, UBE2L3, UBQLN4, UCN, UCN2, USF1, USP4, VDR, YDJC, ZFP36L1, ZFP90, ZGPAT, ZNF300P1, ZNF831, ZPBP, ZPBP2 |
| **Psoriasis (Ps)** | 62 | B3GNT2, CARD14, CARM1, COG6, DDX58, ELMO1, ERAP1, ETS1, EXOC2, FBXL19, FUT2, GJB2, HLA-B, HLA-C, IFIH1, IL4, IL12B, IL13, IL23A, IL23R, ILF3, IRF4, KCNH7, KLF4, LCE3A, LCE3B, LCE3D, MBD2, NFKBIA, NOS2, PRDX5, PRM3, PRSS53, PSMA6, PTRF, REL, RNF114, RPS6KA4, RPS26, RUNX3, SDC4, SLC9A8, SLC45A1, SNAI1, SOCS1, SPATA2, STARD6, STAT2, STAT3, STAT5A, STAT5B, TAGAP, TARBP1, TNFAIP3, TNFRSF9, TNIP1, TRAF3IP2, TSC1, TYK2, UBE2L3, ZC3H12C, ZMIZ1 |
| **Rheumatoid arthritis (RA)** | 138 | ABHD6, ACOXL, AFF3, AHNAK2, AIRE, ANKRD55, ANXA3, APOM, ARAP1, ARHGEF3, ARID5B, ARL15, ATG5, ATM, B3GNT2, BATF, BLK, C1QBP, C5, C5orf30, C6orf10, CASP8, CCL19, CCL21, CCR6, CD2, CD5, CD28, CD40, CD83, CD226, CD247, CDK2, CDK4, CDK6, CEP57, CFLAR, CLNK, COG6, CSF2, CSF3, CTLA4, CXCR5, DNASE1L3, EOMES, ETS1, FADS1, FADS2, FADS3, FCGR2A, FCRL3, FLI1, GATA3, GATSL3, GCH1, GPR125, GRHL2, HLA-DQA1, HLA-DQA2, HLA-DQB1, HLA-DRA, HLA-DRB1, HLA-G, IFNGR2, IGFBP1, IKZF3, IL2, IL2RA, IL2RB, IL3, IL6R, IL6ST, IL20RB, IL21, INPP5B, IRAK1, IRF4, IRF5, IRF8, JAZF1, KCNIP4, KIF5A, LBH, MED1, MICA, MMEL1, MTF1, NFKBIE, OLIG3, P2RY10, PADI4, |

| | | |
|---|---|---|
| | | PDE2A, PFKL, PIP4K2C, PLCL2, PLD4, POU3F1, PPIL4, PRKCH, PRKCQ, PTPN2, PTPN11, PTPN22, PVT1, PXK, RAD51B, RAG1, RAG2, RASGRP1, RBPJ, RCAN1, REL, RPS12P4, RTKN2, RUNX1, SALL3, SFTPD, SH2B3, SPRED2, STAT4, SYNGR1, TAGAP, TEC, TNFAIP3, TNFRSF9, TNFRSF14, TPD52, TRAF1, TRAF6, TRHDE, TXNDC11, TYK2, UBASH3A, UBE2L3, WDFY4, YDJC, ZNF438, ZNF774 |
| **$IMD_4$** | 228 | ADAD1, ADCY3, AFF3, AHSA2, ANKFN1, ANO6, ANTXR2, ASCL2, ATXN2, B3GNT2, BACH2, BAGE, BAGE2, C1QTNF6, C4orf17, C6orf120, CAPSL, CARD14, CARD9, CARM1, CCR1, CCR2, CCR3, CCR4, CCR5, CCR9, CCRL2, CCT8L2, CD226, CD247, CD28, CD69, CD80, CDK2, CENPO, CIITA, CLEC16A, COBL, COG6, CTLA4, CTSH, CUX2, DDX58, DIO3, DLK1, DLL1, DNAJC27, DNMT3A, DOK5, EAPP, EDIL3, EFR3B, ELMO1, EMX2, EMX2OS, EOMES, ERAP1, ERAP2, ERBB3, ETS1, EXOC2, FAM120B, FASLG, FBXL19, FCGR2A, FOSL2, FRMD4B, FUT2, GJB2, GLIS3, GPR25, GPR35, GPR37, GPR65, H19, HAPLN1, HHAT, HLA-A, HLA-B, HLA-C, HLA-DQA1, HLA-DQB1, HLA-DRB1, HYDIN, ICOS, ICOSLG, IFIH1, IGF2, IKZF4, IL10, IL12A, IL12B, IL13, IL18R1, IL18RAP, IL1R1, IL1R2, IL1RL1, IL1RL2, IL2, IL21, IL23A, IL23R, IL27, IL2RA, IL4, IL6R, IL7R, ILF3, INS, IRF4, ITGA4, KCNH7, KIAA1109, KIF21B, KLF4, KLK15, KLK2, KLK3, KLK4, KLKP1, LCE3A, LCE3B, LCE3D, LMO7, LPP, LSP1, LTBR, MAP3K7, MBD2, MEG3, MMEL1, MRPL23, MTTP, NAV1, NFIA, NFKBIA, NKX2-3, NOG, NOS2, NPEPPS, ORMDL3, PARK7, PGM1, PHF10, PHTF1, PLEK, POMC, PPP1R12B, PRDX5, PRKCQ, PRM3, PRSS53, PSMA6, PSMB1, PSMG1, PTGER4, PTPN11, PTPN2, PTPN22, PTPRK, PTRF, RAB11FIP2, RAB5B, RASGRP1, REL, RGS1, RNF114, RPS26, RPS6KA4, RTL1, RUNX3, SCHIP1, SDC4, SH2B3, SLC45A1, SLC9A4, SLC9A8, SNAI1, SNX6, SOCS1, SPATA2, STAC, STARD6, STAT2, STAT3, STAT5A, STAT5B, STK32B, SULT1A1, SUOX, SYT2, SYT8, TAGAP, TARBP1, TBKBP1, TBP, TBX21, TCTE3, TH, THEMIS, TLR7, TLR8, TNFAIP3, TNFRSF11B, TNFRSF14, TNFRSF1A, TNFRSF9, TNFSF18, TNFSF4, TNIP1, TNNI2, TNNT3, TNP2, TPTE, TRAF3IP2, TRAFD1, TSC1, TYK2, UBASH3A, UBE2E3, UBE2L3, UBE2T, WDR27, YDJC, ZC3H12C, ZFP36L1, ZMIZ1 |
| **$IMD_5$** | 331 | ABHD6, ACOXL, ADAD1, ADCY3, AFF3, AHNAK2, AHSA2, AIRE, ANKFN1, ANKRD55, ANO6, ANTXR2, ANXA3, APOM, ARAP1, ARHGEF3, ARID5B, ARL15, ASCL2, ATG5, ATM, ATXN2, B3GNT2, BACH2, BAGE, BAGE2, BATF, BLK, C1QBP, C1QTNF6, C4orf17, C5, C5orf30, C6orf10, C6orf120, CAPSL, CARD14, CARD9, CARM1, CASP8, CCL19, CCL21, CCR1, CCR2, |

| | | |
|---|---|---|
| | | CCR3, CCR4, CCR5, CCR6, CCR9, CCRL2, CCT8L2, CD2, CD226, CD247, CD28, CD40, CD5, CD69, CD80, CD83, CDK2, CDK4, CDK6, CENPO, CEP57, CFLAR, CIITA, CLEC16A, CLNK, COBL, COG6, CSF2, CSF3, CTLA4, CTSH, CUX2, CXCR5, DDX58, DIO3, DLK1, DLL1, DNAJC27, DNASE1L3, DNMT3A, DOK5, EAPP, EDIL3, EFR3B, ELMO1, EMX2, EMX2OS, EOMES, ERAP1, ERAP2, ERBB3, ETS1, EXOC2, FADS1, FADS2, FADS3, FAM120B, FASLG, FBXL19, FCGR2A, FCRL3, FLI1, FOSL2, FRMD4B, FUT2, GATA3, GATSL3, GCH1, GJB2, GLIS3, GPR125, GPR25, GPR35, GPR37, GPR65, GRHL2, H19, HAPLN1, HHAT, HLA-A, HLA-B, HLA-C, HLA-DQA1, HLA-DQA2, HLA-DQB1, HLA-DRA, HLA-DRB1, HLA-G, HYDIN, ICOS, ICOSLG, IFIH1, IFNGR2, IGF2, IGFBP1, IKZF3, IKZF4, IL10, IL12A, IL12B, IL13, IL18R1, IL18RAP, IL1R1, IL1R2, IL1RL1, IL1RL2, IL2, IL20RB, IL21, IL23A, IL23R, IL27, IL2RA, IL2RB, IL3, IL4, IL6R, IL6ST, IL7R, ILF3, INPP5B, INS, IRAK1, IRF4, IRF5, IRF8, ITGA4, JAZF1, KCNH7, KCNIP4, KIAA1109, KIF21B, KIF5A, KLF4, KLK15, KLK2, KLK3, KLK4, KLKP1, LBH, LCE3A, LCE3B, LCE3D, LMO7, LPP, LSP1, LTBR, MAP3K7, MBD2, MED1, MEG3, MICA, MMEL1, MRPL23, MTF1, MTTP, NAV1, NFIA, NFKBIA, NFKBIE, NKX2-3, NOG, NOS2, NPEPPS, OLIG3, ORMDL3, P2RY10, PADI4, PARK7, PDE2A, PFKL, PGM1, PHF10, PHTF1, PIP4K2C, PLCL2, PLD4, PLEK, POMC, POU3F1, PPIL4, PPP1R12B, PRDX5, PRKCH, PRKCQ, PRM3, PRSS53, PSMA6, PSMB1, PSMG1, PTGER4, PTPN11, PTPN2, PTPN22, PTPRK, PTRF, PVT1, PXK, RAB11FIP2, RAB5B, RAD51B, RAG1, RAG2, RASGRP1, RBPJ, RCAN1, REL, RGS1, RNF114, RPS12P4, RPS26, RPS6KA4, RTKN2, RTL1, RUNX1, RUNX3, SALL3, SCHIP1, SDC4, SFTPD, SH2B3, SLC45A1, SLC9A4, SLC9A8, SNAI1, SNX6, SOCS1, SPATA2, SPRED2, STAC, STARD6, STAT2, STAT3, STAT4, STAT5A, STAT5B, STK32B, SULT1A1, SUOX, SYNGR1, SYT2, SYT8, TAGAP, TARBP1, TBKBP1, TBP, TBX21, TCTE3, TEC, TH, THEMIS, TLR7, TLR8, TNFAIP3, TNFRSF11B, TNFRSF14, TNFRSF1A, TNFRSF9, TNFSF18, TNFSF4, TNIP1, TNNI2, TNNT3, TNP2, TPD52, TPTE, TRAF1, TRAF3IP2, TRAF6, TRAFD1, TRHDE, TSC1, TXNDC11, TYK2, UBASH3A, UBE2E3, UBE2L3, UBE2T, WDFY4, WDR27, YDJC, ZC3H12C, ZFP36L1, ZMIZ1, ZNF438, ZNF774 |
| **Type 1 diabetes (T1D)** | 60 | ADCY3, AFF3, BACH2, C1QTNF6, C6orf120, CAPSL, CD69, CD226, CDK2, CENPO, CLEC16A, COBL, CTLA4, CTSH, CUX2, DIO3, DLK1, DLL1, DNAJC27, DNMT3A, EFR3B, ERBB3, FAM120B, FOSL2, GLIS3, HLA-DRB1, IFIH1, IKZF4, IL2, IL2RA, IL7R, IL10, IL27, INS, LMO7, MEG3, ORMDL3, PGM1, PHF10, PHTF1, POMC, PRKCQ, PRM3, PSMB1, PTPN2, PTPN11, |

| | | |
|---|---|---|
| | | PTPN22, RAB5B, RASGRP1, RTL1, SH2B3, SUOX, TBP, TCTE3, TNFRSF11B, TNP2, TRAFD1, TYK2, UBASH3A, WDR27 |
| | | |
| **Prediction Tool** | | |
| **CADD** | 112 | AAMP, ADAD1, ADCY3, AMZ1, ANKRD55, APOBR, ATG16L1, BACH2, C1orf106, CCDC26, CCDC85B, CCDC116, CCL2, CCL7, CD40, CD226, CDKAL1, CISD1, CLN3, CNTF, CPEB4, CREM, CRTC3, CTSW, CUL2, DAP, DENND1B, DNMT3B, ERAP2, FADS1, FADS2, FEN1, FIBP, FOSL1, FOSL2, FUT2, GALC, GNA12, GNB2, GPR65, GPR183, GPX4, HORMAD2, HOTTIP, HOXA11-AS, HOXA13, IFNG-AS1, IFNGR2, IKZF3, IL27, IPMK, IRGM, ITGAL, ITLN1, JAZF1, KIAA0907, KIAA1109, LNPEP, LPXN, LRRK2, MAML2, MIR611, MIR623, MIR3939, MTMR3, MUC19, NDFIP1, NFKB1, NKX2-3, NOTCH2, NR5A2, NXPE1, NXPE2P1, NXPE4, PARK7, PHACTR2, PLCL1, PNKD, PPP5C, PRKCB, PTPN2, PUS10, REV3L, RGS14, RIT1, RMI2, RNU7-77P, RSPO3, SBNO2, SCARNA5, SFMBT1, SKAP2, SLAIN2, SLC22A5, SLC39A11, SMAD3, THADA, THEMIS, TMBIM1, TNFRSF14, TNFSF15, TRAF3IP2-AS1, UBAC2, UBE2L3, YDJC, ZFP36L1, ZFP90, ZFP91, ZFP91-CNTF, ZMIZ1, ZNF300, ZNF300P1 |
| **GWAVA** | 140 | AAMP, ADAM30, ADCY3, AMZ1, ANKRD55, APOBR, ATG16L1, BACH2, C1orf106, C5orf56, C10orf55, CCDC26, CCDC85B, CCDC116, CCL2, CCL7, CD40, CD226, CDC37, CDKAL1, CISD1, CLN3, CNTF, CPEB4, CREM, CTSW, CUL2, CXCR5, DENND1B, DNMT3B, DOK6, EDEM2, FADS1, FADS2, FCAR, FEN1, FIBP, FOSL1, FOSL2, FUT2, GALC, GIGYF1, GNA12, GNB2, GPR65, GPR183, HHEX, HIF3A, HLA-DQA1, HMGB3P4, HORMAD2, HOTTIP, HOXA11-AS, HOXA13, IFIH1, IKZF3, IL2RA, IL10, IL18R1, IL18RAP, IL27, IPMK, IRF5, IRGM, ITGAL, ITLN1, JAK2, JAZF1, KIAA1109, KSR1, LIME1, LINC00511, LINC00598, LNPEP, LPXN, LRRK2, LSP1, MIR611, MIR1181, MIR1908, MIR4772, MTMR3, MUC19, NDFIP1, NKX2-3, NOTCH2, NR5A2, NXPE1, NXPE4, OR5B21, PARK7, PDE4A, PHACTR2, PLAGL2, PLAU, PLCL1, PNKD, PPM1G, PPP5C, PRKCB, PRM1, PRM2, PRM3, PTGIR, PTPN22, RGS14, RIT1, RMI2, RNASET2, RNU7-77P, RSL24D1P6, RSPO3, SBNO2, SKAP2, SLAIN2, SLC2A4RG, SLC22A5, SLC39A11, SMAD3, SNORA48, SP110, SP140, TM9SF4, TMBIM1, TNFRSF14, TNFSF15, TRAF3IP2, TRAF3IP2-AS1, TSPY26P, UBAC2, UBE2L3, YDJC, ZBTB46, ZFP36L1, ZFP90, ZFP91, ZFP91-CNTF, ZGPAT, ZNF300, ZNF831 |
| **RegulomeDB** | 98 | ADCY3, AMZ1, ANKRD55, APOBR, ATG16L1, BACH2, C1orf106, C5orf56, C10orf55, CCDC26, CCDC85B, CCDC116, |

| | | |
|---|---|---|
| | | CCL2, CD40, CDC37, CFL1, CISD1, CLN3, CPEB4, CREM, CTSW, CUL2, CXCR5, DAP, DNMT3B, EFEMP2, ERAP2, FADS1, FADS2, FAM213B, FEN1, FIBP, FOSL1, FOSL2, GNA12, HDAC7, HORMAD2, HOTTIP, HOXA11-AS, HOXA13, IKZF3, IL2RA, IL10, IL27, IPMK, IRF5, IRGM, ITGAL, JAK2, JAZF1, KIAA0907, KSR1, LIME1, LINC00511, LNPEP, LSP1, MIR611, MIR1181, MIR3611, MIR3939, MIR4298, MTMR3, NDFIP1, NFKB1, NKX2-3, PARK7, PDE4A, PLAGL2, PLAU, PLCL1, PNKD, PRKCB, PRM1, PTPN22, REV3L, RGS14, RIT1, RMI2, RNASET2, RNU7-77P, SBNO2, SKAP2, SLC2A4RG, SLC22A5, SMAD3, SNX32, TMBIM1, TNFRSF14, TRAF3IP2-AS1, TSPAN14, TSPY26P, UBE2L3, YDJC, ZBTB46, ZFP90, ZGPAT, ZMIZ1, ZNF300 |
| **CADD+GWAVA** | 58 | AAMP, ANKRD55, APOBR, ATG16L1, BACH2, CCDC116, CCDC26, CCDC85B, CCL2, CCL7, CD40, CDKAL1, CLN3, CNTF, CPEB4, CREM, DENND1B, DNMT3B, FADS1, FADS2, FEN1, FIBP, FOSL1, FOSL2, GALC, GNB2, GPR183, HOTTIP, HOXA11-AS, HOXA13, IKZF3, IL27, ITGAL, LPXN, LRRK2, MIR611, MUC19, NKX2-3, NOTCH2, NR5A2, PHACTR2, PLCL1, PNKD, PPP5C, PRKCB, RNU7-77P, RSPO3, SKAP2, SLAIN2, SLC22A5, SLC39A11, SMAD3, TMBIM1, UBAC2, UBE2L3, YDJC, ZFP91, ZFP91-CNTF |
| **CADD+ RegulomeDB** | 34 | ADCY3, ANKRD55, BACH2, CCDC26, CCDC85B, CD40, CISD1, CPEB4, CREM, DNMT3B, FADS1, FADS2, FEN1, FIBP, FOSL1, IKZF3, JAZF1, KIAA0907, MIR3939, MIR611, NDFIP1, PLCL1, PNKD, PRKCB, REV3L, RIT1, RNU7-77P, SKAP2, SMAD3, TMBIM1, TRAF3IP2-AS1, UBE2L3, YDJC, ZFP90 |
| **GWAVA+ RegulomeDB** | 63 | AMZ1, ANKRD55, APOBR, BACH2, C10orf55, C5orf56, CCDC26, CCDC85B, CCL2, CD40, CDC37, CISD1, CLN3, CPEB4, CREM, CXCR5, DNMT3B, FADS1, FADS2, FEN1, FIBP, FOSL1, FOSL2, GNA12, HORMAD2, HOTTIP, HOXA11-AS, HOXA13, IKZF3, IL10, IL27, IL2RA, IPMK, IRF5, IRGM, ITGAL, JAK2, KSR1, LINC00511, LSP1, MIR1181, MIR611, MTMR3, NDFIP1, NKX2-3, PDE4A, PLAU, PLCL1, PNKD, PRKCB, PTPN22, RGS14, RMI2, RNASET2, RNU7-77P, SBNO2, SKAP2, SMAD3, TMBIM1, UBE2L3, YDJC, ZFP90, ZNF300 |
| **CADD+GWAVA+ RegulomeDB** | 20 | ANKRD55, BACH2, CCDC26, CCDC85B, CD40, CREM, DNMT3B, FADS1, FADS2, FEN1, FIBP, FOSL1, IKZF3, MIR611, PLCL1, PRKCB, RNU7-77P, SMAD3, UBE2L3, YDJC |
