# Supplementary material for: In-Silico Analysis of Inflammatory Bowel Disease (IBD) GWAS Loci to Novel Connections
Source: PLoS One. 2015 Mar 18;10(3):e0119420. doi: 10.1371/journal.pone.0119420 (PMC4364731; doi:10.1371/journal.pone.0119420)
Supplement: S4 Table — (PDF) [file pone.0119420.s004.pdf]

S4 Table. Summary of the gene overlap between the reference disease and the prediction tool.

Prediction Tool	Number of overlapping genes							
	**AS (43)**	**CeD (100)**	**IBD (297)**	**PS (62)**	**RA (138)**	**T1D (60)**	**IMD_4 (228)**	**IMD_5 (331)**
**CADD (112)**	7	11	61	3	12	6	19	27
**GWAVA (140)**	5	11	71	5	15	10	14	32
**RegulomeDB (98)**	6	7	54	2	13	7	15	23
**CADD+GWAVA (58)**	4	3	30	1	7	3	6	11
**CADD+RegulomeDB (34)**	2	3	22	1	8	2	4	10
**GWAVA+RegulomeDB (63)**	4	4	36	1	11	6	10	17
**CADD+GWAVA+RegulomeDB (20)**	2	3	12	1	7	1	3	8
