# Supplementary material for: In-Silico Analysis of Inflammatory Bowel Disease (IBD) GWAS Loci to Novel Connections
Source: PLoS One. 2015 Mar 18;10(3):e0119420. doi: 10.1371/journal.pone.0119420 (PMC4364731; doi:10.1371/journal.pone.0119420)
Supplement: S1 File — (PDF) [file pone.0119420.s005.pdf]

S1 File: Enrichment scores for AS, CeD, IBD, Ps, RA, T1D, IMD_4 and IMD_5 are presented in line-graphs in Figure A – Figure H, respectively.

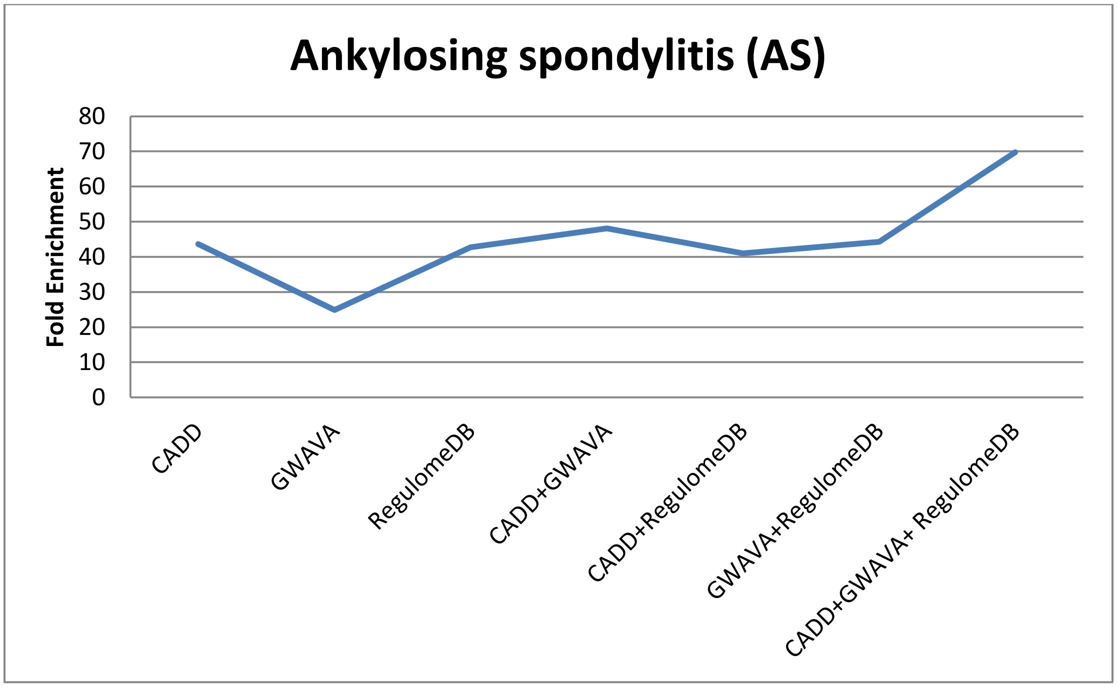

Figure A. Enrichment scores for ankylosing spondylitis (AS).

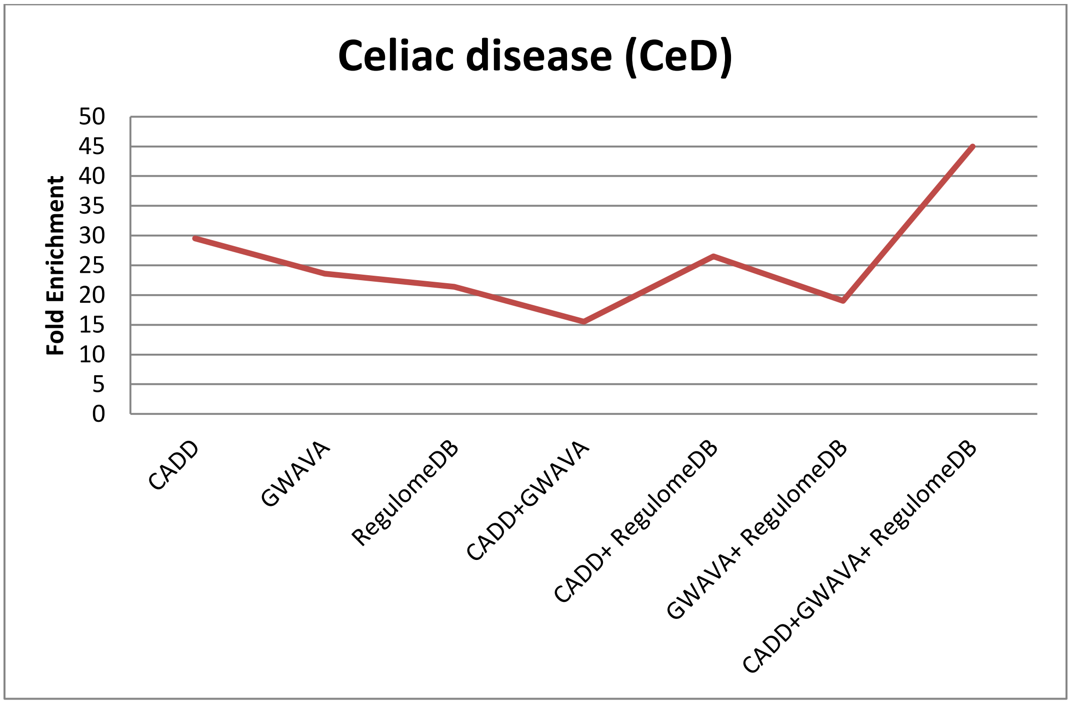

Figure B. Enrichment scores for celiac disease (CeD).

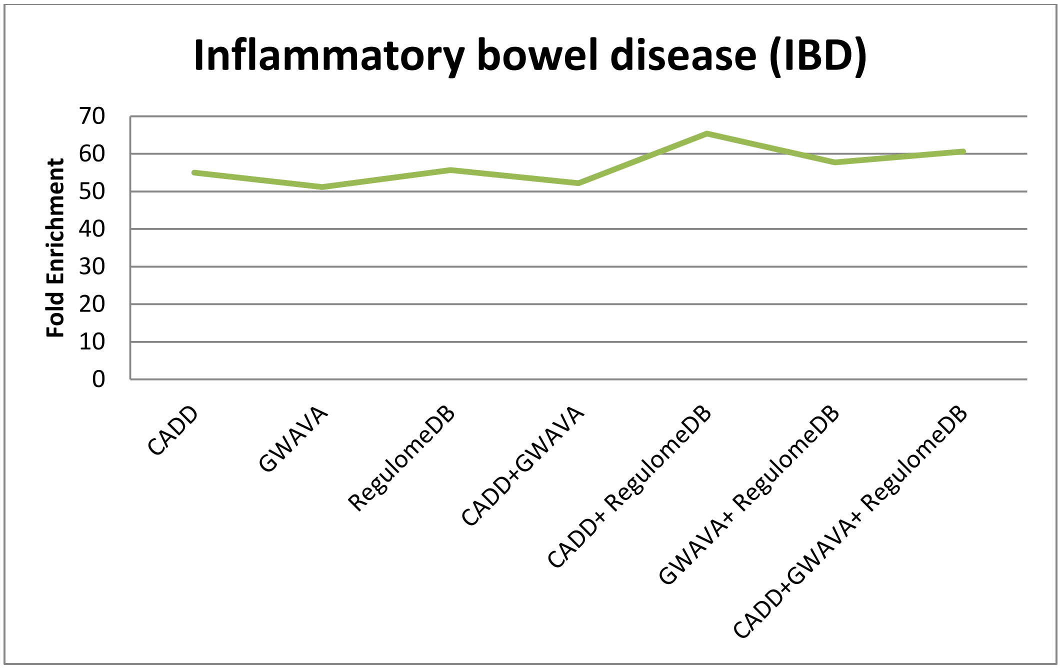

Figure C. Enrichment scores for inflammatory bowel disease (IBD).

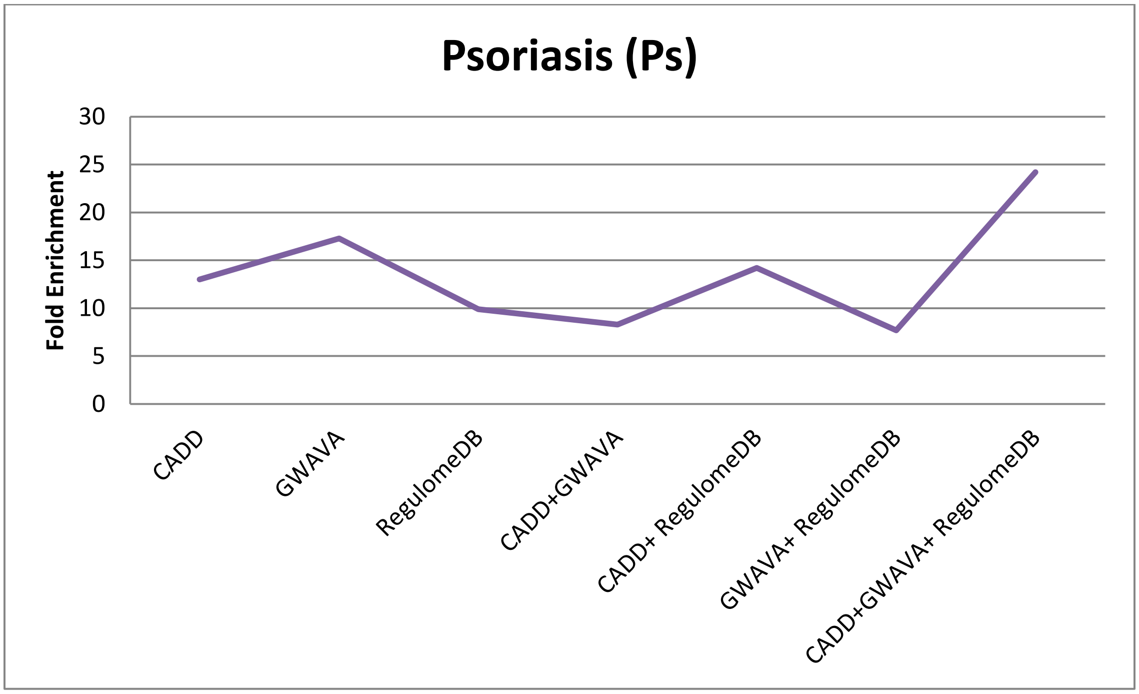

Figure D. Enrichment scores for psoriasis (Ps).

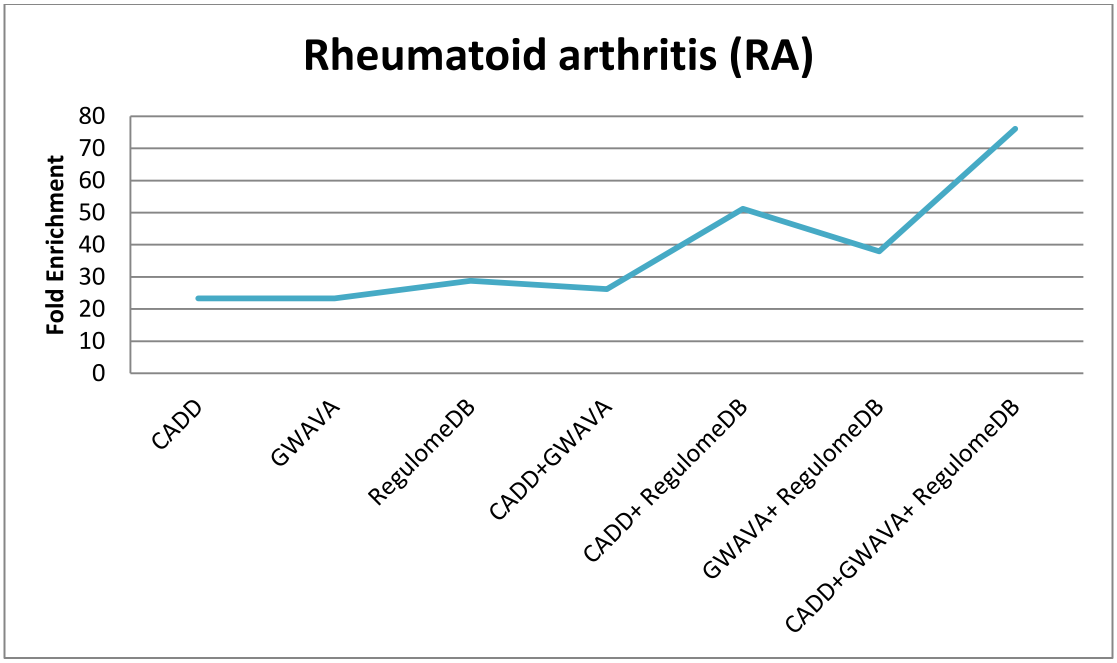

Figure E. Enrichment scores for rheumatoid arthritis (RA).

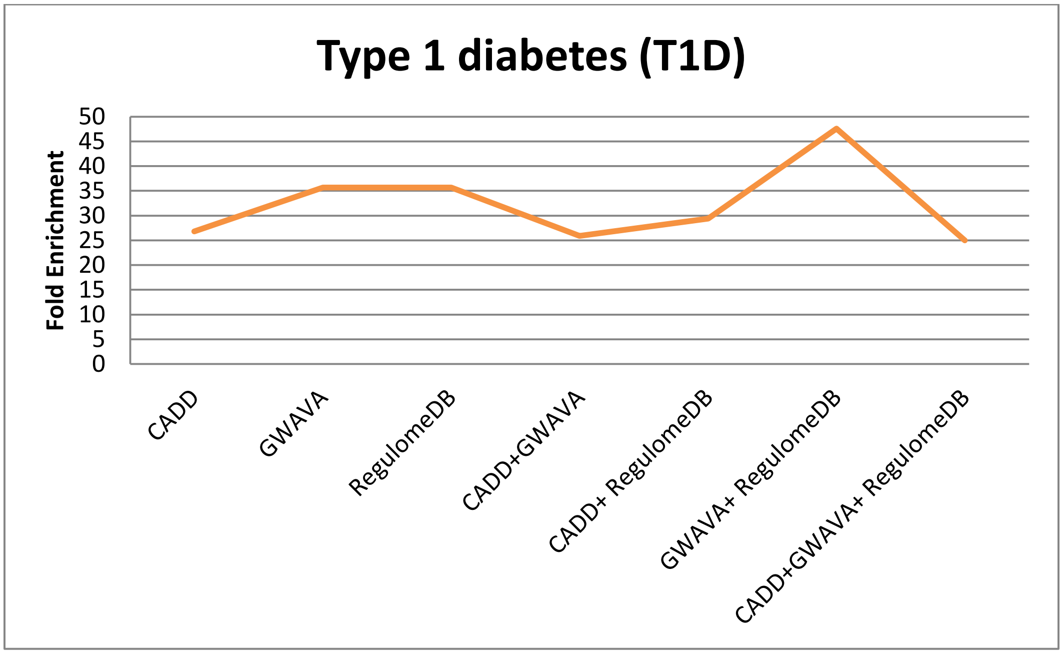

Figure F. Enrichment scores for type 1 diabetes (T1D).

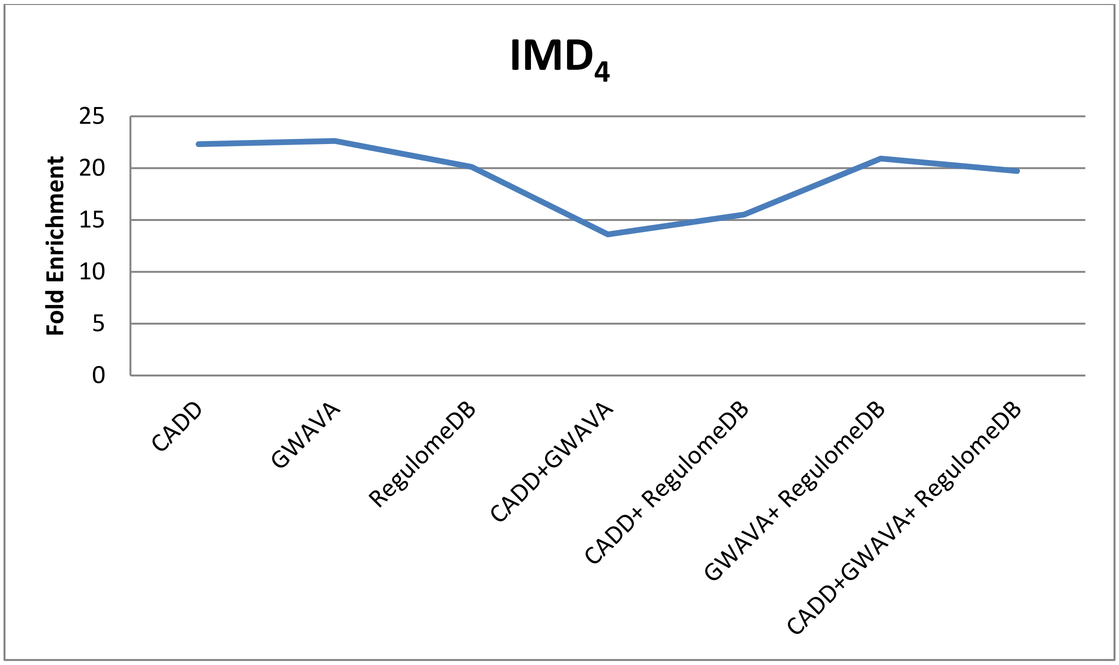

Figure G. Fold Enrichment for IMD_4 (AS+CeD+Ps+T1D).

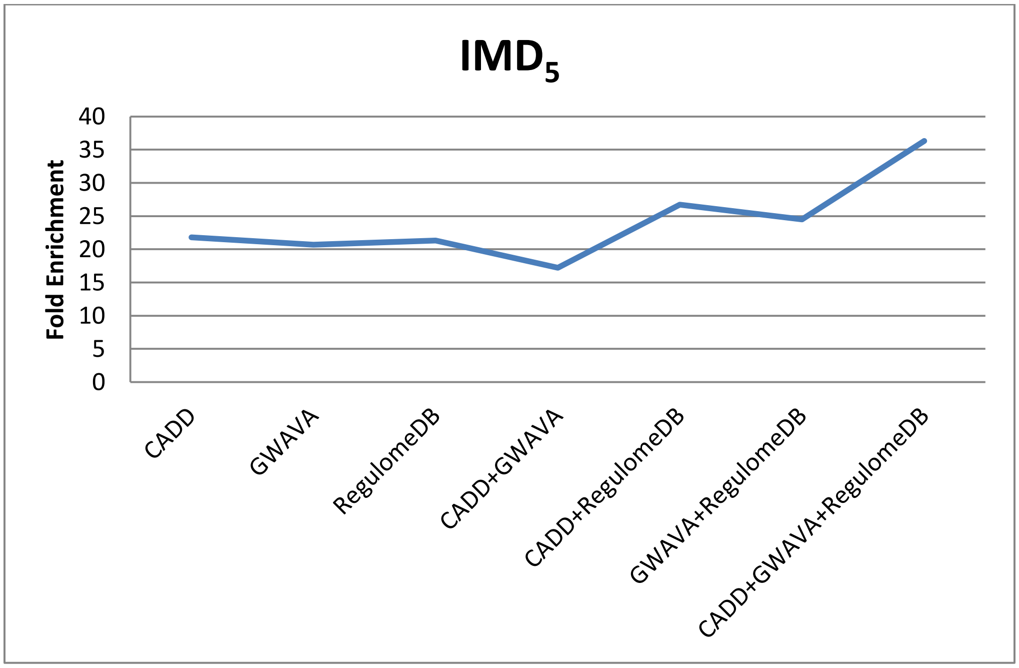

Figure H. Enrichment scores for IMD_5 (AS+CeD+Ps+RA+T1D).
